# Fermatean Fuzzy Schweizer–Sklar Operators and BWM-Entropy-Based Combined Compromise Solution Approach: An Application to Green Supplier Selection

**DOI:** 10.3390/e24060776

**Published:** 2022-05-31

**Authors:** Dongmei Wei, Dan Meng, Yuan Rong, Yi Liu, Harish Garg, Dragan Pamucar

**Affiliations:** 1 School of Computer and Software Engineering, Xihua University, Chengdu 610039, China; wdm@mail.xhu.edu.cn; 2 School of Computing and Artificial Intelligence, Southwestern University of Finance and Economics, Chengdu 611130, China; 3 School of Management Science and Engineering, Southwestern University of Finance and Economics, Chengdu 611130, China; mengd_t@swufe.edu.cn; 4 School of Management, Shanghai University, Shanghai 200444, China; 5 Data Recovery Key Laboratory of Sichuan Province, Neijiang Normal University, Neijiang 641100, China; liuyi@njtc.edu.cn; 6 School of Mathematics, Thapar Institute of Engineering and Technology (Deemed University), Patiala 147004, India; harish.garg@thapar.edu; 7 Department of Logistics, University of Defence in Belgrade, 11000 Belgrade, Serbia; dragan.pamucar@va.mod.gov.rs

**Keywords:** green supplier selection, Fermatean fuzzy set, Schweizer–Sklar, entropy, CoCoSo method

## Abstract

The Fermatean fuzzy set (FFS) is a momentous generalization of a intuitionistic fuzzy set and a Pythagorean fuzzy set that can more accurately portray the complex vague information of elements and has stronger expert flexibility during decision analysis. The Combined Compromise Solution (CoCoSo) approach is a powerful decision-making technique to choose the ideal objective by fusing three aggregation strategies. In this paper, an integrated, multi-criteria group-decision-making (MCGDM) approach based on CoCoSo and FFS is used to assess green suppliers. To begin, several innovative operations of Fermatean fuzzy numbers based on Schweizer–Sklar norms are presented, and four aggregation operators utilizing the proposed operations are also developed. Several worthwhile properties of the advanced operations and operators are explored in detail. Next, a new Fermatean fuzzy entropy measure is propounded to determine the combined weight of criteria, in which the subjective and objective weights are computed by an improved best-and-worst method (BWM) and entropy weight approach, respectively. Furthermore, MCGDM based on CoCoSo and BWM-Entropy is brought forward and employed to sort diverse green suppliers. Lastly, the usefulness and effectiveness of the presented methodology is validated by comparison, and the stability of the developed MCGDM approach is shown by sensitivity analysis. The results shows that the introduced method is more stable during ranking of green suppliers, and the comparative results expound that the proposed method has higher universality and credibility than prior Fermatean fuzzy approaches.

## 1. Introduction

As a key part of enterprise operation and management, green supplier selection not only has a direct impact on the quality and cost control of enterprise products, but also is conducive to the sustainable development of the circular economy and green economy. Because green supplier selection needs to involve multiple criteria with different dimensions and expert groups and suppliers with different qualifications, green supplier selection is usually regarded as a complex, multi-criteria group-decision-making process. In recent years, scholars have studied the selection of green suppliers under different uncertainties and decision algorithms [1,2,3,4,5,6]. Wu et al. [7] constructed an integrated green-supplier-selection group-decision model through combining BWM and VIKOR under an interval type-2 environment. Liang et al. [8] introduced a hybrid green-supplier-selection decision algorithm on the basis of alternative queuing method (AQM) and linguistic Z-numbers to take into account the credibility of experts’ preferences, where step-weight assessment ratio analysis (SWARA) is utilized to determine the importance of the considered criteria. Gao et al. [9] suggested an innovative probabilistic linguistic consensus decision framework based on the consensus measure and feedback mechanism to choose the optimal green supplier. Ma et al. [10] brought a three-way group decision methodology by extending a decision–theoretic rough set into hesitant fuzzy linguistics to evaluate the most-satisfying supplier. Further, in order to comprehensively analyze the literature on green supplier selection, Zhang et al. [11] developed a comprehensive overview of green supplier evaluation and selection through summarizing and analyzing the research from 2009 to 2020 and providing some novel research directions and challenges. By considering the psychological behavior of experts during decision analysis, Zhang et al. [12] advanced a spherical fuzzy MABAC approach based on cumulative prospect theory for choosing a suitable green supplier. Wang et al. [13] presented probabilistic dual hesitant fuzzy BWM and superiority and inferiority ranking (SIR) based on reaching consistency in order to select the proper suppliers. The mentioned research is based on diverse complicated uncertainty models, but it fails to solve the problems with complex fuzzy linguistic information. Therefore, Krishankumar et al. [14] put forward a more universal green-supplier-selection group-decision approach based on case-based and TODIM methods with double-hierarchy hesitant fuzzy linguistic information.

Multi-criteria group decision-making in modern decision science integrates multiple disciplines and is based on the evaluation of information obtained by experts from their cognitive abilities and preferences and adopts scientific decision-making methods with multiple qualitative or quantitative criteria to select the ideal goal from the target set. Because of its significant merits in management decision analysis, it has been extensively employed in different fields, such as sustainable development, low-carbon energy, engineering construction and so forth. Nevertheless, the complexity of objective things and limitations of human cognition bring lots of challenges when experts give their assessments or preferences with respect to the considered criteria. Fortunately, a fuzzy set (FS) [15], originated by Zadeh, can validly manage this kind of phenomenon and has achieved numerous positive outcomes. Furthermore, intuitionistic FSs [16] describe fuzzy information more comprehensively by adding non-membership degree and hesitation degree, with the sum of membership degree and non-membership grade (NMG) being no greater than one. Thereafter, in order to provide more options for experts to express their judgment or opinion with the help of membership grade (MG) and NMG, Yager [17,18] proposed the concept of Pythagorean FSs by expanding the limiting conditions of membership and non-membership such that the sum of squares of MG and NMG is no more than 1. Since PFS can provide more assessment options to express a greater number of expert opinions, it has proven an efficient model for experts to portray the vagueness and incomplete information of realistic complex problems. In light of the superiority of PFS in addressing uncertainty, much research using PFS has been developed to settle diverse complex, real-word decision issues [19,20,21,22,23,24,25,26].

Recently, a novel spread of intuitionistic and Pythagorean FS called Fermatean fuzzy set [27,28] was pioneered as a powerful tool to describe the indeterminacy and ambiguity of actual MCGDM problems. FFS also portrays uncertainty evaluations of objectives by MG and NMG and makes their cubic sums less than or equal to one. Due to its practicability and applicability, many scholars focus on it and attain many enriching theoretical results and practical application. Senapati and Yager [28] introduced a Fermatean fuzzy WPM decision method with the help of several novel operations and operators to choose a satisfactory bridge construction. In order to make the information aggregation more flexible, TOPSIS is extended to Fermatean fuzzy sets on the basis of some novel Dombi operators [29]. Mishra and Rani [30] constructed a group-decision model based on entropy, score function and WASPAS to select the best location for a healthcare waste-disposal center. Further, Gül [31] determined a satisfactory COVID-19 testing laboratory by extended SAW, ARAS and VIKOR with the Fermatean fuzzy method. However, those extensions of FFS fail to compute weight during decision analysis. Mishra et al. [32] presented an innovative generalized score function of FFS and combined CRITIC and EDAS to build a sustainable third-party reverse logistics provider assessment algorithm, where expert weight and criteria importance are computed by generalized score function and CRITIC. Thereafter, to enhance the robustness and reliability of decision-analysis outcomes, Rani and Mishra [33] propounded an improved MULTIMOORA approach with the aid of Fermatean fuzzy Einstein weight averaging and geometric operators to select a suitable electric vehicle charging station. In addition, a Fermatean fuzzy CRITIC–COPRAS approach has been proffered to manage the challenges of sustainable digital transformation [34]. Apart from the aforementioned investigations, several extensions of FFS by considering different application environments have been presented to enrich the representation of uncertainty. Liu et al. [35,36] originated the Fermatean fuzzy linguistic set and proposed the corresponding TODIM and TOPSIS approaches by linguistic scale functions-based novel distance measures. In order to more exactly express expert preferences, Jeevaraj [37] defined interval-valued FFS and discussed the related order, score and distance measures that laid the foundation of decision model construction. Further, Mishra et al. [38] built up the COPRAS method with extended interval-valued hesitant Fermatean fuzzy theories in order to choose an optimal desalination technology. Luo and Liu [39] presented hesitant FFS and constructed a novel regional green development assessment model by combining hesitant FFS and MULTIMOORA.

The aggregation operator is a vital tool to integrate the assessment information of multiple experts during MCGDM. In order to aggregate Fermatean fuzzy information, many aggregation operators have been defined on the basis of different Archimedean operations. Senapati and Yager [40] first defined the basic operations of Fermatean fuzzy numbers, introduced several corresponding operators, and gave an MCDM decision algorithm by using the proposed operators. Garg et al. [41] presented some novel Fermatean fuzzy Yager operators to build a flexible decision algorithm for choosing an optimal lab for COVID-19 testing. Shahzadi et al. [42] defined the Hamacher interactive operations of FFN and developed Fermatean fuzzy Hamacher interactive weighted averaging operators. Further, Shit and Ghorai [43] put forward some Fermatean fuzzy Dombi operators to aggregate FFNs and built an MCDM method based on them. The above Fermatean fuzzy operators were obtained by using different operations of Archimedean t-norm and t-conorm and can validly fuse Fermatean fuzzy information. In addition, as a particular case of Archimedean t-norm and t-conorm, Schweizer–Sklar operations [44] not only generate the intersection and union of FFS but also possess a parameter to flexibly adjust the operations. Since its introduction, it has been extended to different fuzzy environments to construct the associated aggregation operators [45,46,47,48]. Zindani et al. [49] proffered a novel group-decision method by merging Schweizer–Sklar power operators and TODIM with inter-valued intuitionistic fuzzy circumstances. Liu et al. [50] defined the Schweizer–Sklar operations of complex q-rung orthopair fuzzy numbers and proposed different Muirhead mean operators to determine interrelations between multiple input data. To further strengthen the practicability of MULTIMOORA, the picture fuzzy Schweizer–Sklar operators-based MULTIMOORA algorithm was presented for group decision analysis [51]. Nevertheless, there is no investigation on Schweizer–Sklar operations for FFS and use of it to build aggregation operators.

In the past few years, many classical decision approaches have been presented to deal with vague and imprecise practical issues. Recently, an excellent decision technique called the Combined Compromise Solution method was proposed by Yazdani et al. [52] to obtain more comprehensive and robust alternatives with the aid of three fusion strategies. As a utility-based decision method, it acquires the final compromise result from multiple angles and utilizes an integrated function to aggregate multiple solutions, which further strengthens the reliability and stability of the ultimate decision outcomes. Owing to its advantages of simple operation and high flexibility, CoCoSo has been extended to different uncertainty environments and used for practical problem evalutaion [53,54,55]. Rani and Mishra [56] established a group decision-making model with completely unknown expert and attribute weight based on similarity and CoCoSo by improving the similarity of single-valued intelligent sets. Yazdani et al. [57] proposed a complete consistency model of rough sets to determine the subjective weight of attributes and combined it with data envelopment analysis, establishing a comprehensive CoCoSo method with rough sets in order to select a satisfactory logistics center. Wang and Wang [58] propounded an integrated linguistic terms with weakened hedges qualitative CoCoSo group assessment framework to evaluate health-care waste treatment technologies. Yet, to the best of our knowledge, extant research does not combine the Schweizer–Sklar operators, entropy and CoCoSo for supplier selection using Fermatean fuzzy sets.

### 1.1. Motivations of This Research

Based on the discussion and literature review, it is apparent that FFS possesses a stronger uncertain information representation efficiency than IFS and PFS in dealing with complex and indeterminacy decision problems. Although much research using FFS has successfully provided support for decision analysis, some novel aggregation theory, information measures and decision techniques need to be explored for experts to analyze real decision issues more comprehensively. From the existing literature, the motivations of this study can be outlined as follows:

(1) Aggregation-based decision algorithms provide a simple and fast manner for experts to comprehensively assess alternatives. Hence, proposing some reasonable and flexible aggregation operator is necessary to integrate Fermatean fuzzy information. The Schweizer–Sklar operations can not only generate operations of FFN but also possess an alternate parameter to make the decision analysis procedure more flexible.
(2) The importance of criteria in decision analysis is very vital for acquiring rational decisions. However, most Fermatean fuzzy decision methodologies only consider the objective weight of criteria but ignore the importance of subjective preferences of criteria produced by experts. Therefore, it is necessary to construe a synthesized criteria-weight-determination model to get more accurate results.
(3) Existing decision approaches using Fermatean fuzzy environments to rank alternatives fail to consider multiple fusion strategies, which will lead to inaccurate decisions. Further, ranking different aggregation strategies is also vital for the final decision result. Hence, it is essential to take multiple fusion strategies and their rankings into account to achieve more robust results.

### 1.2. Contributions of This Research

In view of the mentioned motivations and discussion of the extant research, the main objective of this study is to propose an integrated Fermatean fuzzy group-decision approach by combining Schweizer–Sklar operations, BWM, entropy and CoCoSo to assess a green supplier. Fermatean fuzzy Schweizer–Sklar operators are employed to aggregate expert evaluations and improve the classical CoCoSo model. Entropy is utilized to determine the objective weight and extend BWM to a Fermatean fuzzy setting. Accordingly, the contributions of this investigation are as below:

♠ Several novel Fermatean fuzzy aggregation operators, such as Fermatean fuzzy Schweizer–Sklar weighted averaging operator, Fermatean fuzzy Schweizer–Sklar weighted geometric operator and corresponding ordered weighted forms are brought forward on the basis of Fermatean fuzzy Schweizer–Sklar operational laws to fuse Fermatean fuzzy information; the related desirable properties of the propounded Fermatean fuzzy operators are also explored at length;
♠ A novel Fermatean fuzzy information entropy measure is proffered to measure the vagueness of FFS and further used to compute the objective weight of criteria.
♠ A compositional weight determination model is constructed based on entropy weight and BWM-entropy to more reasonably identify the weight information of criteria;
♠ An integrated Fermatean fuzzy group decision framework is built in light of the proposed Fermatean fuzzy Schweizer–Sklar operators, combined weight determination model and improved CoCoSo to address complicated decision issues with unknown weight information.

### 1.3. Structure of This Research

To meet the objectives of our study, the remainder of the paper is arranged as follows: Section 2 succinctly reviews background information related to this paper. Section 3 propounds a novel Fermatean fuzzy entropy measure to determine the weight of criteria. In Section 4, some Fermatean fuzzy Schweizer–Sklar weighted averaging and geometric operators are presented; also, some properties are discussed. Section 5 constructs an integrated Fermatean fuzzy CoCoSo group-decision framework for selecting a satisfactory supplier. In Section 6, a supplier assessment problem is utilized to show feasibility, and a contrastive study is implemented to highlight the merits of the developed method. Several conclusion remarks are listed at the end.

## 2. Preliminaries

This section reviews several basic concepts, such as FFS and Schweizer–Sklar t-conorm and t-norm, that will be utilized to build our decision approach.

### 2.1. FFS

The FFS was originally proposed to represent uncertain information more effectively than intuitionistic FS and Pythagorean FS. In what follows, we illustrate the definition and operations of FFS [27].

**Definition** **1** (Ref. [27])**.** *Assume Y is a domain of discourse. A Fermatean fuzzy set (FFS) F on Y is represented as*

$$\begin{array}{c} F=\{\langle Y,\phi_{F}\left(y\right),\psi_{F}\left(y\right)\rangle |y\in Y\} \end{array}$$

*where $\phi_{F}\left(y\right)$ and $\psi_{F}\left(y\right)$ signify the grade of membership and non-membership of element y to Y, with the restriction that $0\leq {\left(\phi_{F}\left(y\right)\right)}^{3}+{\left(\psi_{F}\left(y\right)\right)}^{3}\leq 1$. The pair $F=\left(\phi_{F}\left(y\right),\psi_{F}\left(y\right)\right)$ is usually utilized to signify a Fermatean fuzzy number (FFN) and simplified as $F=\left(\phi_{F},\psi_{F}\right)$ with $0\leq \phi_{F}^{3}+\psi_{F}^{3}\leq 1$. The hesitancy grade of y belongs to F$\pi_{F}\left(y\right)=\sqrt[{3}]{1-{\left(\phi_{F}\right)}^{3}-{\left(\psi_{F}\right)}^{3}}$.*

**Definition** **2** (Ref. [27])**.** *Let $F_{1}=\left(\phi_{F_{1}},\psi_{F_{1}}\right)$ and $F_{2}=\left(\phi_{F_{2}},\psi_{F_{2}}\right)$ be two FFNs, then the operational laws deduced on by algebraic operations are:*

$$\begin{array}{cc} (1)\; & F_{1}⊕F_{2}=\left(\sqrt[{3}]{1-\left(1-{\left(\phi_{F_{1}}\right)}^{3}\right)\left(1-{\left(\phi_{F_{2}}\right)}^{3}\right)},\psi_{F_{1}}\psi_{F_{2}}\right);\\ (2)\; & F_{1}⊗F_{2}=\left(\phi_{F_{1}}\phi_{F_{2}},\sqrt[{3}]{1-\left(1-{\left(\psi_{F_{1}}\right)}^{3}\right)\left(1-{\left(\psi_{F_{2}}\right)}^{3}\right)}\right);\\ (3)\; & \lambda \cdot F_{1}=\left(\sqrt[{3}]{1-{\left(1-{\left(\phi_{F_{1}}\right)}^{3}\right)}^{\lambda }},{\left(\psi_{F_{1}}\right)}^{\lambda }\right),\lambda >0;\\ (4)\; & F_{1}^{\lambda }=\left({\left(\phi_{F_{1}}\right)}^{\lambda },\sqrt[{3}]{1-{\left(1-{\left(\varphi_{F_{1}}\right)}^{3}\right)}^{\lambda }}\right),\lambda >0;\\ (5)\; & F_{1}^{c}=\left(\psi_{F_{1}},\phi_{F_{1}}\right). \end{array}$$

In order to compare two FFNs, Senapati and Yager [27] proposed the score function $SC\left(F\right)={\left(\phi_{F}\right)}^{3}-{\left(\psi_{F}\right)}^{3}$ and accuracy function $AC\left(F\right)={\left(\phi_{F}\right)}^{3}+{\left(\psi_{F}\right)}^{3}$ to compare FFNs according to their score and accuracy values, respectively. However, the score function SC(F) is invalid when the membership grade is equal to the non-membership grade. Based on this, a novel score function S(F) is presented by [30] to consider the influence of hesitancy grade for enhancing the reasonableness of comparison and ranking.

**Definition** **3** (Ref. [30])**.** *Given an FFN $F=\left(\phi_{F},\psi_{F}\right)$, the score function S(F) of F is defined as:*

(1) $$\begin{array}{c} S\left(F\right)=\frac{1}{2}\left(\left({\left(\phi_{F}\right)}^{3}-{\left(\psi_{F}\right)}^{3}-\ln\left(1+{\left(\pi_{F}\right)}^{3}\right)\right)+1\right),\;S\left(F\right)\in \left[0,1\right], \end{array}$$

*where $\pi_{F}=\sqrt[{3}]{1-{\left(\phi_{F}\right)}^{3}-{\left(\psi_{F}\right)}^{3}}$ signifies the hesitancy grade of F.*

**Definition** **4.** *Let $F_{1}=\left(\phi_{F_{1}},\psi_{F_{1}}\right)$ and $F_{2}=\left(\phi_{F_{2}},\psi_{F_{2}}\right)$ be two FFNs. Then the comparison algorithm of $F_{1}$ and $F_{2}$ is:*

 *(1)*  *If $S\left(F_{1}\right)<S\left(F_{2}\right)$, then $F_{1}$ is smaller than $F_{2}$, signified as $F_{1}≺F_{2}$;*
 *(2)*  *If $S\left(F_{1}\right)=S\left(F_{2}\right)$, then we need to compare their accuracy values:*
  • *If $AC\left(F_{1}\right)>AC\left(F_{2}\right)$, then $F_{1}$ is bigger than $F_{2}$, signified as $F_{1}≻F_{2}$;*
  • *If $AC\left(F_{1}\right)=AC\left(F_{2}\right)$, then $F_{1}$ has no differences with $F_{2}$, signified as $F_{1}∼F_{2}$.*

### 2.2. Schweizer–Sklar T-Conorm and T-Norm

The Schweizer–Sklar t-conorm and t-norm, consisting of the Schweizer–Sklar product and Schweizer–Sklar sum, respectively, are special cases of ATT.

**Definition** **5** (Ref. [44]). *Suppose $F_{1}=\left(\phi_{F_{1}},\psi_{F_{1}}\right)$ and $F_{2}=\left(\phi_{F_{2}},\psi_{F_{2}}\right)$ are two FFNs. Then the generalized intersection and union are described as follows:*

(2) $$\begin{array}{cc} \; & F_{1}\cap_{\tilde{T},{\tilde{T}}^{*}}F_{2}=\left\{\langle y,\tilde{T}\left(\phi_{F_{1}},\phi_{F_{2}}\right),{\tilde{T}}^{*}\left(\psi_{F_{1}},\psi_{F_{2}}\right)\rangle y\in Y\right\} \end{array}$$

(3) $$\begin{array}{cc} \; & F_{1}\cup_{\tilde{T},{\tilde{T}}^{*}}F_{2}=\left\{\langle y,{\tilde{T}}^{*}\left(\phi_{F_{1}},\phi_{F_{2}}\right),\tilde{T}\left(\psi_{F_{1}},\psi_{F_{2}}\right)\rangle y\in Y\right\} \end{array}$$

*where $\tilde{T}$ represents a T-norm and ${\tilde{T}}^{*}$ represents a t-conorm. The definitions of the Schweizer–Sklar t-norm and t-conorm are shown as follows.*

Let a,b be two positive real numbers and a,b∈[0,1]. Then the conception of the Schweizer–Sklar t-norm and t-conorm is depicted as follows:

$$\begin{array}{cc} \; & {\tilde{T}}_{SS,\sigma }\left(a,b\right)={\left(a^{\sigma }+b^{\sigma }-1\right)}^{\frac{1}{\sigma }},\sigma <0;\\ \; & {\tilde{T}}_{SS,\sigma }^{*}\left(a,b\right)=1-{\left({\left(1-a\right)}^{\sigma }+{\left(1-b\right)}^{r}-1\right)}^{\frac{1}{\sigma }},\sigma <0. \end{array}$$

Specially, when σ=0, the Schweizer–Sklar t-norm and t-conorm shall yield to the algebraic t-norm and t-conorm, namely, ${\tilde{T}}_{\sigma }\left(a,b\right)=ab$, ${\tilde{T}}_{\sigma }^{*}\left(a,b\right)=a+b-ab$.

## 3. A Novel Fermatean Fuzzy Entropy Measure

Entropy is a frequently used and valid information measure for measuring the fuzziness of FS. Since the conception of fuzzy entropy was presented, diverse information entropies have been introduced under intuitionistic FS, Pythagorean FS and hesitant FS. These entropy measures not only enrich the information measure theory of FS, but are also widely used in decision analysis [59,60,61,62]. Hence, this section proposes a novel Fermatean fuzzy entropy measure to measure the vagueness of FFS, and uses it to determine the weight of assessment criteria.

**Definition** **6** (Ref. [30])**.** *A real-value mapping E: $FFS\left(Y\right)\to \left[0,1\right]$ is a Fermatean fuzzy entropy measure if it meets the following conditions:*

 *(P1)*  *$0\leq E\left(F\right)\leq 1$;*
 *(P2)*  *$E\left(F\right)=0$ if F is a crisp set;*
 *(P3)*  *$E\left(F\right)=1\Rightarrow \phi_{F}\left(y_{t}\right)=\psi_{F}\left(y_{t}\right)$ for $y_{t}\in Y$;*
 *(P4)*  *$E\left(F\right)=E\left({\left(F\right)}^{c}\right)$;*
 *(P5)*  *$E\left(F\right)\leq E\left(G\right)$ for all $F,G\in FFS\left(Y\right)$ meet either if $\phi_{F}\left(y_{t}\right)\leq \phi_{G}\left(y_{t}\right)\leq \psi_{G}\left(y_{t}\right)\leq \psi_{F}\left(y_{t}\right)$ or $\phi_{F}\left(y_{t}\right)\geq \phi_{G}\left(y_{t}\right)\geq \psi_{G}\left(y_{t}\right)\geq \psi_{F}\left(y_{t}\right)$ for all $y_{t}\in Y$.*

**Definition** **7.** *For an FFS $F=\{\langle Y,\phi_{F}\left(y\right),\psi_{F}\left(y\right)\rangle |y\in Y\}$, the Fermatean fuzzy entropy of F is defined as:*

(4) $$\begin{array}{c} E\left(F\right)=\frac{1}{2n}\sum_{t=1}^{n}\left[\frac{e^{-\left|{\left(\phi_{F}\left(y_{t}\right)\right)}^{3}-{\left(\psi_{F}\left(y_{t}\right)\right)}^{3}\right|}-e^{-1}}{1-e^{-1}}+\left(1-\left|{\left(\phi_{F}\left(y_{t}\right)\right)}^{3}-{\left(\psi_{F}\left(y_{t}\right)\right)}^{3}\right|\right)\right]. \end{array}$$

**Proof.**

(P1) Since $0\leq \phi_{F}\left(y_{t}\right),\psi_{F}\left(y_{t}\right)\leq 1$, then $0\leq {\left(\phi_{F}\left(y_{t}\right)\right)}^{3},{\left(\psi_{F}\left(y_{t}\right)\right)}^{3}\leq 1$. Thus, we have $0\leq \left|{\left(\phi_{F}\left(y_{t}\right)\right)}^{3}-{\left(\psi_{F}\left(y_{t}\right)\right)}^{3}\right|\leq 1$. Let $f\left(a\right)=\frac{e^{-a}-e^{-1}}{1-e^{-1}}+\left(1-a\right)\left(0\leq a\leq 1\right)$, then $f^{{}^{\prime }}\left(a\right)=-\frac{e^{-a}}{1-e^{-1}}-1<0$, namely, $f\left(a\right)$ is decreasing in [0,1]. Thus one has $0\leq f\left(a\right)\leq 2$. Further, $0\leq \frac{1}{2n}\sum_{t=1}^{n}\left[\frac{e^{-\left|{\left(\phi_{F}\left(y_{t}\right)\right)}^{3}-{\left(\psi_{F}\left(y_{t}\right)\right)}^{3}\right|}-e^{-1}}{1-e^{-1}}+\left(1-\left|{\left(\phi_{F}\left(y_{t}\right)\right)}^{3}-{\left(\psi_{F}\left(y_{t}\right)\right)}^{3}\right|\right)\right]\leq 1$. Accordingly, $0\leq E\left(F\right)\leq 1$ holds.
(P2) If $E\left(F\right)=0$, then $\left[\frac{e^{-\left|{\left(\phi_{F}\left(y_{t}\right)\right)}^{3}-{\left(\psi_{F}\left(y_{t}\right)\right)}^{3}\right|}-e^{-1}}{1-e^{-1}}+\left(1-\left|{\left(\phi_{F}\left(y_{t}\right)\right)}^{3}-{\left(\psi_{F}\left(y_{t}\right)\right)}^{3}\right|\right)\right]=0$. This implies that $\left|{\left(\phi_{F}\left(y_{t}\right)\right)}^{3}-{\left(\psi_{F}\left(y_{t}\right)\right)}^{3}\right|=1$, thus $\phi_{F}\left(y_{t}\right)=1,\psi_{F}\left(y_{t}\right)=0$ or $\phi_{F}\left(y_{t}\right)=0,\psi_{F}\left(y_{t}\right)=1$. We prove that F is a crisp set. Conversely, if F is a crisp set, $\phi_{F}\left(y_{t}\right)=1,\psi_{F}\left(y_{t}\right)=0$ or $\phi_{F}\left(y_{t}\right)=0,\psi_{F}\left(y_{t}\right)=1$. Based on Equation (4), we have $E\left(F\right)=0$.
(P3) If $\phi_{F}\left(y_{t}\right)=\psi_{F}\left(y_{t}\right)$ for $y_{t}\in Y$, we get $E\left(F\right)=1$ with the aid of Equation (4). Conversely, if $E\left(F\right)=1$, then $\forall y_{t}\in Y$; we have $\frac{e^{-\left|{\left(\phi_{F}\left(y_{t}\right)\right)}^{3}-{\left(\psi_{F}\left(y_{t}\right)\right)}^{3}\right|}-e^{-1}}{1-e^{-1}}=1$ and $\left(1-\left|{\left(\phi_{F}\left(y_{t}\right)\right)}^{3}-{\left(\psi_{F}\left(y_{t}\right)\right)}^{3}\right|\right)=1$; then $\left|{\left(\phi_{F}\left(y_{t}\right)\right)}^{3}-{\left(\psi_{F}\left(y_{t}\right)\right)}^{3}\right|=0$, namely, $\phi_{F}\left(y_{t}\right)=\psi_{F}\left(y_{t}\right)$.
(P4) Since ${\left(F\right)}^{c}$ is the complement of FFS F, then ${\left(F\right)}^{c}=\left\{y_{t},\psi_{F}\left(y_{t}\right),\phi_{F}\left(y_{t}\right)|y_{t}\in Y\right\}$. Now we can obtain
  $$\begin{array}{cc} E\left({\left(F\right)}^{c}\right) & =\frac{1}{2n}\sum_{t=1}^{n}\left[\frac{e^{-\left|{\left(\psi_{F}\left(y_{t}\right)\right)}^{3}-{\left(\phi_{F}\left(y_{t}\right)\right)}^{3}\right|}-e^{-1}}{1-e^{-1}}+\left(1-\left|{\left(\psi_{F}\left(y_{t}\right)\right)}^{3}-{\left(\phi_{F}\left(y_{t}\right)\right)}^{3}\right|\right)\right]\\ \; & =\frac{1}{2n}\sum_{t=1}^{n}\left[\frac{e^{-\left|{\left(\phi_{F}\left(y_{t}\right)\right)}^{3}-{\left(\psi_{F}\left(y_{t}\right)\right)}^{3}\right|}-e^{-1}}{1-e^{-1}}+\left(1-\left|{\left(\phi_{F}\left(y_{t}\right)\right)}^{3}-{\left(\psi_{F}\left(y_{t}\right)\right)}^{3}\right|\right)\right]=E\left(F\right). \end{array}$$
  Hence, $E\left(F\right)=E\left({\left(F\right)}^{c}\right)$ holds for all $y_{t}\in Y$.
(P5) All $y_{t}\in Y$ meet if either $\phi_{F}\left(y_{t}\right)\leq \phi_{G}\left(y_{t}\right)\leq \psi_{G}\left(y_{t}\right)\leq \psi_{F}\left(y_{t}\right)$ or $\phi_{F}\left(y_{t}\right)\geq \phi_{G}\left(y_{t}\right)\geq \psi_{G}\left(y_{t}\right)\geq \psi_{F}\left(y_{t}\right)$; then $-\left|{\left(\phi_{F}\left(y_{t}\right)\right)}^{3}-{\left(\psi_{F}\left(y_{t}\right)\right)}^{3}\right|\leq -\left|{\left(\phi_{G}\left(y_{t}\right)\right)}^{3}-{\left(\psi_{G}\left(y_{t}\right)\right)}^{3}\right|$ holds for all $y_{t}\in Y$. This implies that $E\left(F\right)\leq E\left(G\right)$ for all $F,G\in FFS\left(Y\right)$ meet if either $\phi_{F}\left(y_{t}\right)\leq \phi_{G}\left(y_{t}\right)\leq \psi_{G}\left(y_{t}\right)\leq \psi_{F}\left(y_{t}\right)$ or $\phi_{F}\left(y_{t}\right)\geq \phi_{G}\left(y_{t}\right)\geq \psi_{G}\left(y_{t}\right)\geq \psi_{F}\left(y_{t}\right)$ for all $y_{t}\in Y$.

 □

## 4. Fermatean Fuzzy Schweizer–Sklar Aggregation Operators

This section first presents the Fermatean fuzzy Schweizer–Sklar operations and then advances some novel Fermatean fuzzy Schweizer–Sklar aggregation operators; also, some valuable properties are explored.

### 4.1. Fermatean Fuzzy Schweizer–Sklar Operations

In light of the ${\tilde{T}}_{SS,\sigma }\left(a,b\right)$ and ${\tilde{T}}_{\sigma }^{*}\left(a,b\right)$ of Schweizer–Sklar operations, the generalized intersection and union operations, respectively, of FFNs are defined as:

**Definition** **8.** *Suppose $F_{1}=\left(\phi_{F_{1}},\psi_{F_{1}}\right)$ and $F_{2}=\left(\phi_{F_{2}},\psi_{F_{2}}\right)$ are two FFNs. Then the generalized intersection and union are described as:*

(5) $$\begin{array}{cc} \; & F_{1}{⊕}_{\tilde{T},{\tilde{T}}^{*}}F_{2}=\left(\sqrt[{3}]{{\tilde{T}}_{SS,\sigma }^{*}\left({\left(\phi_{F_{1}}\right)}^{3},{\left(\phi_{F_{2}}\right)}^{3}\right)},\sqrt[{3}]{{\tilde{T}}_{SS,\sigma }\left({\left(\psi_{F_{1}}\right)}^{3},{\left(\psi_{F_{2}}\right)}^{3}\right)}\right), \end{array}$$

(6) $$\begin{array}{cc} \; & F_{1}{⊗}_{\tilde{T},{\tilde{T}}^{*}}F_{2}=\left(\sqrt[{3}]{{\tilde{T}}_{SS,\sigma }\left({\left(\phi_{F_{1}}\right)}^{3},{\left(\phi_{F_{2}}\right)}^{3}\right)},\sqrt[{3}]{{\tilde{T}}_{SS,\sigma }^{*}\left({\left(\psi_{F_{1}}\right)}^{3},{\left(\psi_{F_{2}}\right)}^{3}\right)}\right). \end{array}$$

In view of Definition 7, we can propound the following operation laws of FFNs:

(7) $$\begin{array}{cc} \; & F_{1}{⊕}_{SS}F_{2}=\left(\sqrt[{3}]{1-{\left({\left(1-{\left(\phi_{F_{1}}\right)}^{3}\right)}^{\sigma }+{\left(1-{\left(\phi_{F_{2}}\right)}^{3}\right)}^{\sigma }-1\right)}^{\frac{1}{\sigma }}},\sqrt[{3}]{{\left({\left(\psi_{F_{1}}\right)}^{3\sigma }+{\left(\psi_{F_{2}}\right)}^{3\sigma }-1\right)}^{\frac{1}{\sigma }}}\right); \end{array}$$

(8) $$\begin{array}{cc} \; & F_{1}{⊗}_{SS}F_{2}=\left(\sqrt[{3}]{{\left({\left(\phi_{F_{1}}\right)}^{3\sigma }+{\left(\phi_{F_{2}}\right)}^{3\sigma }-1\right)}^{\frac{1}{\sigma }}},\sqrt[{3}]{1-{\left({\left(1-{\left(\psi_{F_{1}}\right)}^{3}\right)}^{\sigma }+{\left(1-{\left(\psi_{F_{2}}\right)}^{3}\right)}^{\sigma }-1\right)}^{\frac{1}{\sigma }}}\right); \end{array}$$

(9) $$\begin{array}{cc} \; & \kappa F_{1}=\left(\sqrt[{3}]{1-{\left(\kappa {\left(1-{\left(\phi_{F_{1}}\right)}^{3}\right)}^{\kappa }-\left(\kappa -1\right)\right)}^{\frac{1}{\kappa }}},\sqrt[{3}]{{\left(\kappa {\left(\psi_{F_{1}}\right)}^{3\sigma }-\left(\kappa -1\right)\right)}^{\frac{1}{\sigma }}}\right); \end{array}$$

(10) $$\begin{array}{cc} \; & F_{1}^{\kappa }=\left(\sqrt[{3}]{{\left(\kappa {\left(\phi_{F_{1}}\right)}^{3\sigma }-\left(\kappa -1\right)\right)}^{\frac{1}{\sigma }}},\sqrt[{3}]{1-{\left(\kappa {\left(1-{\left(\psi_{F_{1}}\right)}^{3}\right)}^{\kappa }-\left(\kappa -1\right)\right)}^{\frac{1}{\kappa }}}\right). \end{array}$$

**Theorem** **1.** *Suppose $F_{1}=\left(\phi_{F_{1}},\psi_{F_{1}}\right)$ and $F_{2}=\left(\phi_{F_{2}},\psi_{F_{2}}\right)$ are two FFNs, and $\kappa ,\kappa_{1},\kappa_{2}>0$. Then*

$$\begin{array}{cc} (1)\; & F_{1}{⊕}_{SS}F_{2}=F_{2}{⊕}_{SS}F_{1};\\ (2)\; & F_{1}{⊗}_{SS}F_{2}=F_{2}{⊗}_{SS}F_{1};\\ (3)\; & \kappa \left(F_{1}{⊕}_{SS}F_{2}\right)=\kappa F_{1}{⊕}_{SS}\kappa F_{2};\\ (4)\; & \kappa_{1}F_{1}{⊕}_{SS}\kappa_{2}F_{1}=\left(\kappa_{1}+\kappa_{2}\right)F_{1};\\ (5)\; & F_{1}^{\kappa_{1}}{⊗}_{SS}F_{1}^{\kappa_{2}}=F_{1}^{\kappa_{1}+\kappa_{2}};\\ (6)\; & {\left(F_{1}\right)}^{\kappa_{1}}{⊗}_{SS}{\left(F_{2}\right)}^{\kappa_{1}}={\left(F_{1}⊗F_{2}\right)}^{\kappa_{1}}. \end{array}$$

The proof of Theorem 1 is straightforward.

### 4.2. Fermatean Fuzzy Schweizer–Sklar Weighted Averaging Operator

In this section, we present the Fermatean fuzzy Schweizer–Sklar weighted averaging (FFSSWA) operator and Fermatean fuzzy Schweizer–Sklar order-weighted averaging (FFSSOWA) operator and explore some of their notable properties.

**Definition** **9.** *Suppose $F_{t}=\left(\phi_{F_{t}},\psi_{F_{t}}\right)$ is a family of FFNs; the FFSSWA operator is a mapping from $\Theta^{n}$ to Θ. If*

(11) $$\begin{array}{c} FFSSWA\left(F_{1},F_{2},\cdots ,F_{n}\right)=\varsigma_{1}F_{1}{⊕}_{SS}\varsigma_{2}F_{2}{⊕}_{SS}\cdots {⊕}_{SS}\varsigma_{n}F_{n}, \end{array}$$

*then the FFSSWA is called a Fermatean fuzzy Schweizer–Sklar weighted averaging operator, where *Θ* signifies the set of FFNs and $\varsigma_{t}\left(t=1\left(1\right)n\right)$ is the weight of $F_{t}$ with $\varsigma_{t}\in \left[0,1\right]$ with $\sum_{t=1}^{n}\varsigma_{t}=1$. Moreover, an FFSSWA operator will yield to an FFSSA operator when $\varpi_{t}={\left(\frac{1}{n},\frac{1}{n},\cdots ,\frac{1}{n}\right)}^{T}$.*

The following theorem can be attained on the basis of Definition 8.

**Theorem** **2.** *Suppose $F_{t}=\left(\phi_{F_{t}},\psi_{F_{t}}\right)$ is a family of FFNs. Then the fusion value through employing the FFSSWA operator is still an FFN, and is represented as*

(12) $$\begin{array}{c} FFSSWA\left(F_{1},F_{2},\cdots ,F_{n}\right)=\left(\begin{array}{c} \sqrt[{3}]{1-{\left(\sum_{t=1}^{n}\varsigma_{t}{\left(1-{\left(\phi_{F_{t}}\right)}^{3}\right)}^{\sigma }-\sum_{t=1}^{n}\varsigma_{t}+1\right)}^{\frac{1}{\sigma }}},\\ \sqrt[{3}]{{\left(\sum_{t=1}^{n}\varsigma_{t}{\left(\phi_{F_{t}}\right)}^{3\sigma }-\sum_{t=1}^{n}\varsigma_{t}+1\right)}^{\frac{1}{\sigma }}} \end{array}\right). \end{array}$$

Based on the mathematical induction method, we can prove that Equation (12) is valid.

In view of the operational laws of FFNs based on Schweizer–Sklar operations, one has

$$\begin{array}{c} \varsigma_{t}F_{t}=\left(\sqrt[{3}]{1-{\left(\varsigma_{t}{\left(1-{\left(\phi_{F_{t}}\right)}^{3}\right)}^{\sigma }-\left(\varsigma_{t}-1\right)\right)}^{\frac{1}{\sigma }}},\sqrt[{3}]{{\left(\varsigma_{t}{\left(\psi_{F_{t}}\right)}^{3\sigma }-\left(\varsigma_{t}-1\right)\right)}^{\frac{1}{\sigma }}}\right). \end{array}$$

(i) When n=2, we have
  $$\begin{array}{cc} \; & \varsigma_{1}F_{1}=\left(\sqrt[{3}]{1-{\left(\varsigma_{1}{\left(1-{\left(\phi_{F_{1}}\right)}^{3}\right)}^{\sigma }-\left(\varsigma_{1}-1\right)\right)}^{\frac{1}{\sigma }}},\sqrt[{3}]{{\left(\varsigma_{1}{\left(\psi_{F_{1}}\right)}^{3\sigma }-\left(\varsigma_{1}-1\right)\right)}^{\frac{1}{\sigma }}}\right);\\ \; & \varsigma_{2}F_{2}=\left(\sqrt[{3}]{1-{\left(\varsigma_{2}{\left(1-{\left(\phi_{F_{2}}\right)}^{3}\right)}^{\sigma }-\left(\varsigma_{2}-1\right)\right)}^{\frac{1}{\sigma }}},\sqrt[{3}]{{\left(\varsigma_{2}{\left(\psi_{F_{2}}\right)}^{3\sigma }-\left(\varsigma_{2}-1\right)\right)}^{\frac{1}{\sigma }}}\right). \end{array}$$
  Then
  $$\begin{array}{cc} \; & FFSSWA\left(F_{1},F_{2}\right)=\varsigma_{1}F_{1}{⊕}_{SS}\varsigma_{2}F_{2}\\ \; & =\left(\begin{array}{c} \sqrt[{3}]{1-{\left({\left(1-{\left(\sqrt[{3}]{1-{\left(\varsigma_{1}{\left(1-{\left(\phi_{F_{1}}\right)}^{3}\right)}^{\sigma }-\left(\varsigma_{1}-1\right)\right)}^{\frac{1}{\sigma }}}\right)}^{3}\right)}^{\sigma }+{\left(1-{\left(\sqrt[{3}]{1-{\left(\varsigma_{2}{\left(1-{\left(\phi_{F_{2}}\right)}^{3}\right)}^{\sigma }-\left(\varsigma_{2}-1\right)\right)}^{\frac{1}{\sigma }}}\right)}^{3}\right)}^{\sigma }-1\right)}^{\frac{1}{\sigma }}},\\ \sqrt[{3}]{{\left({\left(\sqrt[{3}]{{\left(\varsigma_{1}{\left(\psi_{F_{1}}\right)}^{3\sigma }-\left(\varsigma_{1}-1\right)\right)}^{\frac{1}{\sigma }}}\right)}^{3\sigma }+{\left(\sqrt[{3}]{{\left(\varsigma_{2}{\left(\psi_{F_{2}}\right)}^{3\sigma }-\left(\varsigma_{2}-1\right)\right)}^{\frac{1}{\sigma }}}\right)}^{3\sigma }-1\right)}^{\frac{1}{\sigma }}} \end{array}\right)\\ \; & =\left(\sqrt[{3}]{1-{\left(\sum_{t=1}^{2}\varsigma_{t}{\left(1-{\left(\phi_{F_{t}}\right)}^{3}\right)}^{\sigma }-\sum_{t=1}^{2}\varsigma_{t}+1\right)}^{\frac{1}{\sigma }}},\sqrt[{3}]{{\left(\sum_{t=1}^{2}\varsigma_{t}{\left(\phi_{F_{t}}\right)}^{3\sigma }-\sum_{t=1}^{2}\varsigma_{t}+1\right)}^{\frac{1}{\sigma }}}\right), \end{array}$$
  Namely, Equation (12) holds for n=2.
(ii) Assume Equation (12) holds n=ℏ.
  $$\begin{array}{c} FFSSWA\left(F_{1},F_{2},\cdots ,F_{\hbar }\right)=\left(\begin{array}{c} \sqrt[{3}]{1-{\left(\sum_{t=1}^{\hbar }\varsigma_{t}{\left(1-{\left(\phi_{F_{t}}\right)}^{3}\right)}^{\sigma }-\sum_{t=1}^{\hbar }\varsigma_{t}+1\right)}^{\frac{1}{\sigma }}},\\ \sqrt[{3}]{{\left(\sum_{t=1}^{\hbar }\varsigma_{t}{\left(\psi_{F_{t}}\right)}^{3\sigma }-\sum_{t=1}^{\hbar }\varsigma_{t}+1\right)}^{\frac{1}{\sigma }}} \end{array}\right). \end{array}$$
  Then when n=ℏ+1, based on the operation rules of FFNs based upon Schweizer–Sklar operations, one has
  $$\begin{array}{c} \varsigma_{\hbar +1}F_{\hbar +1}=\left(\begin{array}{c} \sqrt[{3}]{1-{\left(\varsigma_{\hbar +1}{\left(1-{\left(\phi_{F_{\hbar +1}}\right)}^{3}\right)}^{\sigma }-\left(\varsigma_{\hbar +1}-1\right)\right)}^{\frac{1}{\sigma }}},\\ \sqrt[{3}]{{\left(\varsigma_{\hbar +1}{\left(\psi_{F_{\hbar +1}}\right)}^{3\sigma }-\left(\varsigma_{\hbar +1}-1\right)\right)}^{\frac{1}{\sigma }}} \end{array}\right), \end{array}$$
  and
  $$\begin{array}{cc} \; & FFSSWA\left(F_{1},F_{2},\cdots ,F_{\hbar },F_{\hbar +1}\right)=FFSSWA\left(F_{1},F_{2},\cdots ,F_{\hbar }\right){⊕}_{SS}\varsigma_{\hbar +1}F_{\hbar +1}\\ \; & =\left(\sqrt[{3}]{1-{\left(\sum_{t=1}^{\hbar }\varsigma_{t}{\left(1-{\left(\phi_{F_{t}}\right)}^{3}\right)}^{\sigma }-\sum_{t=1}^{\hbar }\varsigma_{t}+1\right)}^{\frac{1}{\sigma }}},\sqrt[{3}]{{\left(\sum_{t=1}^{\hbar }\varsigma_{t}{\left(\psi_{F_{t}}\right)}^{3\sigma }-\sum_{t=1}^{\hbar }\varsigma_{t}+1\right)}^{\frac{1}{\sigma }}}\right)\\ \; & {⊕}_{SS}\left(\sqrt[{3}]{1-{\left(\varsigma_{\hbar +1}{\left(1-{\left(\phi_{F_{\hbar +1}}\right)}^{3}\right)}^{\sigma }-\left(\varsigma_{\hbar +1}-1\right)\right)}^{\frac{1}{\sigma }}},\sqrt[{3}]{{\left(\varsigma_{\hbar +1}{\left(\psi_{F_{\hbar +1}}\right)}^{3\sigma }-\left(\varsigma_{\hbar +1}-1\right)\right)}^{\frac{1}{\sigma }}}\right)\\ \; & =\left(\sqrt[{3}]{1-{\left(\sum_{t=1}^{\hbar +1}\varsigma_{t}{\left(1-{\left(\phi_{F_{t}}\right)}^{3}\right)}^{\sigma }-\sum_{t=1}^{\hbar +1}\varsigma_{t}+1\right)}^{\frac{1}{\sigma }}},\sqrt[{3}]{{\left(\sum_{t=1}^{\hbar +1}\varsigma_{t}{\left(\psi_{F_{t}}\right)}^{3\sigma }-\sum_{t=1}^{\hbar +1}\varsigma_{t}+1\right)}^{\frac{1}{\sigma }}}\right) \end{array}$$

Accordingly, Equation (12) is valid for n=ℏ+1.

Based on (i) and (ii), Equation (12) holds for any *t*. Furthermore, because $\varsigma_{t}$ is the weight of FFN $F_{t}$ and meets $\varsigma_{t}\in \left[0,1\right]$ with $\sum_{t=1}^{n}\varsigma_{t}=1$, Equation (12) can be simplified as

(13) $$\begin{array}{c} FFSSWA\left(F_{1},F_{2},\cdots ,F_{n}\right)=\left(\sqrt[{3}]{1-{\left(\sum_{t=1}^{n}\varsigma_{t}{\left(1-{\left(\phi_{F_{t}}\right)}^{3}\right)}^{\sigma }\right)}^{\frac{1}{\sigma }}},\sqrt[{3}]{{\left(\sum_{t=1}^{n}\varsigma_{t}{\left(\psi_{F_{t}}\right)}^{3\sigma }\right)}^{\frac{1}{\sigma }}}\right). \end{array}$$

In what follows, we will explore several notable properties of FFSSWA operators.

**Property** **1** (Idempotency)**.** *Suppose $F_{t}=\left(\phi_{F_{t}},\psi_{F_{t}}\right)$ is a family of FFNs. If $F_{t}=\left(\phi_{F},\psi_{F}\right)=F$ for all $F_{t}$, then*

(14) $$\begin{array}{c} FFSSWA\left(F_{1},F_{2},\cdots ,F_{n}\right)=F \end{array}$$

**Proof.** 
$$\begin{array}{cc} FFSSWA\left(F_{1},F_{2},\cdots ,F_{n}\right)\; & =\left(\sqrt[{3}]{1-{\left(\sum_{t=1}^{n}\varsigma_{t}{\left(1-{\left(\phi_{F_{t}}\right)}^{3}\right)}^{\sigma }\right)}^{\frac{1}{\sigma }}},\sqrt[{3}]{{\left(\sum_{t=1}^{n}\varsigma_{t}{\left(\psi_{F_{t}}\right)}^{3\sigma }\right)}^{\frac{1}{\sigma }}}\right)\\ \; & =\left(\sqrt[{3}]{1-{\left({\left(1-{\left(\phi_{F}\right)}^{3}\right)}^{\sigma }\right)}^{\frac{1}{\sigma }}},\sqrt[{3}]{{\left({\left(\psi_{F}\right)}^{3\sigma }\right)}^{\frac{1}{\sigma }}}\right)\\ \; & =\left(\sqrt[{3}]{1-\left(1-{\left(\phi_{F}\right)}^{3}\right)},\sqrt[{3}]{{\left(\psi_{F}\right)}^{3}}\right)\\ \; & =\left(\phi_{F},\phi_{F}\right)=F. \end{array}$$

This concludes the proof of Property 1.  □

**Property** **2** (Boundedness)**.** *Suppose $F_{t}=\left(\phi_{F_{t}},\psi_{F_{t}}\right)$ is a family of FFNs. If $F^{+}=(\max_{1\leq t\leq n}\phi_{F_{t}},$$\min_{1\leq t\leq n}\psi_{F_{t}})$ and $F^{-}=\left(\min_{1\leq t\leq n}\phi_{F_{t}},\max_{1\leq t\leq n}\psi_{F_{t}}\right)$, then*

(15) $$\begin{array}{c} F^{-}\leq FFSSWA\left(F_{1},F_{2},\cdots ,F_{n}\right)\leq F^{+}. \end{array}$$

**Proof.**  Since $\min_{1\leq t\leq n}\phi_{F_{t}}\leq \phi_{F_{t}}\leq \max_{1\leq t\leq n}\phi_{F_{t}}$ and $\min_{1\leq t\leq n}\psi_{F_{t}}\leq \phi_{F_{t}}\leq \max_{1\leq t\leq n}\psi_{F_{t}}$ hold for all *t*. Then we can acquire:

(i) For membership grade of $FFSSWA\left(F_{1},F_{2},\cdots ,F_{n}\right)$, one has
  $$\begin{array}{cc} \; & \;1-{\left(\max_{1\leq t\leq n}\phi_{F_{t}}\right)}^{3}\leq 1-{\left(\phi_{F_{t}}\right)}^{3}\leq 1-{\left(\min_{1\leq t\leq n}\phi_{F_{t}}\right)}^{3}\\ \; & \Rightarrow {\left(1-{\left(\max_{1\leq t\leq n}\phi_{F_{t}}\right)}^{3}\right)}^{\sigma }\leq {\left(1-{\left(\phi_{F_{t}}\right)}^{3}\right)}^{\sigma }\leq {\left(1-{\left(\min_{1\leq t\leq n}\phi_{F_{t}}\right)}^{3}\right)}^{\sigma }\\ \; & \Rightarrow {\left(\sum_{t=1}^{n}\varsigma_{t}{\left(1-{\left(\max_{1\leq t\leq n}\phi_{F_{t}}\right)}^{3}\right)}^{\sigma }\right)}^{\frac{1}{\sigma }}\leq {\left(\sum_{t=1}^{n}\varsigma_{t}{\left(1-{\left(\phi_{F_{t}}\right)}^{3}\right)}^{\sigma }\right)}^{\frac{1}{\sigma }}\leq {\left(\sum_{t=1}^{n}\varsigma_{t}{\left(1-{\left(\min_{1\leq t\leq n}\phi_{F_{t}}\right)}^{3}\right)}^{\sigma }\right)}^{\frac{1}{\sigma }}\\ \; & \Rightarrow \sqrt[{3}]{1-{\left(\sum_{t=1}^{n}\varsigma_{t}{\left(1-{\left(\min_{1\leq t\leq n}\phi_{F_{t}}\right)}^{3}\right)}^{\sigma }\right)}^{\frac{1}{\sigma }}}\leq \sqrt[{3}]{1-{\left(\sum_{t=1}^{n}\varsigma_{t}{\left(1-{\left(\phi_{F_{t}}\right)}^{3}\right)}^{\sigma }\right)}^{\frac{1}{\sigma }}}\leq \sqrt[{3}]{1-{\left(\sum_{t=1}^{n}\varsigma_{t}{\left(1-{\left(\max_{1\leq t\leq n}\phi_{F_{t}}\right)}^{3}\right)}^{\sigma }\right)}^{\frac{1}{\sigma }}}\\ \; & \Rightarrow \min_{1\leq t\leq n}\phi_{F_{t}}\leq \sqrt[{3}]{1-{\left(\sum_{t=1}^{n}\varsigma_{t}{\left(1-{\left(\phi_{F_{t}}\right)}^{3}\right)}^{\sigma }\right)}^{\frac{1}{\sigma }}}\leq \max_{1\leq t\leq n}\psi_{F_{t}}. \end{array}$$
(ii) For non-membership grade of $FFSSWA\left(F_{1},F_{2},\cdots ,F_{n}\right)$, one has
  $$\begin{array}{cc} \; & \;\min_{1\leq t\leq n}\psi_{F_{t}}\leq \psi_{F_{t}}\leq \max_{1\leq t\leq n}\psi_{F_{t}}\\ \; & \Rightarrow \varsigma_{t}{\left(\min_{1\leq t\leq n}\psi_{F_{t}}\right)}^{3\sigma }\leq \varsigma_{t}{\left(\psi_{F_{t}}\right)}^{3\sigma }\leq \varsigma_{t}{\left(\psi_{F_{t}}\leq \max_{1\leq t\leq n}\psi_{F_{t}}\right)}^{3\sigma }\\ \; & \Rightarrow {\left(\sum_{t=1}^{n}\varsigma_{t}{\left(\min_{1\leq t\leq n}\psi_{F_{t}}\right)}^{3\sigma }\right)}^{\frac{1}{\sigma }}\leq {\left(\sum_{t=1}^{n}\varsigma_{t}{\left(\psi_{F_{t}}\right)}^{3\sigma }\right)}^{\frac{1}{\sigma }}\leq {\left(\sum_{t=1}^{n}\varsigma_{t}{\left(\max_{1\leq t\leq n}\psi_{F_{t}}\right)}^{3\sigma }\right)}^{\frac{1}{\sigma }}\\ \; & \Rightarrow \sqrt[{3}]{{\left(\sum_{t=1}^{n}\varsigma_{t}{\left(\min_{1\leq t\leq n}\psi_{F_{t}}\right)}^{3\sigma }\right)}^{\frac{1}{\sigma }}}\leq \sqrt[{3}]{{\left(\sum_{t=1}^{n}\varsigma_{t}{\left(\psi_{F_{t}}\right)}^{3\sigma }\right)}^{\frac{1}{\sigma }}}\leq \sqrt[{3}]{{\left(\sum_{t=1}^{n}\varsigma_{t}{\left(\max_{1\leq t\leq n}\psi_{F_{t}}\right)}^{3\sigma }\right)}^{\frac{1}{\sigma }}}\\ \; & \Rightarrow \min_{1\leq t\leq n}\psi_{F_{t}}\leq \sqrt[{3}]{{\left(\sum_{t=1}^{n}\varsigma_{t}{\left(\psi_{F_{t}}\right)}^{3\sigma }\right)}^{\frac{1}{\sigma }}}\leq \min_{1\leq t\leq n}\psi_{F_{t}}. \end{array}$$
(iii) Furthermore, considering that as the score of the FFSSWA operator, let $FFSSWA\left(F_{1},F_{2},\cdots ,F_{n}\right)=F=\left(\phi_{F},\psi_{F}\right)$. We can acquire
  $$\begin{array}{cc} \; & SC\left(F\right)={\left(\phi_{F}\right)}^{3}-{\left(\psi_{F}\right)}^{3}={\left(\sqrt[{3}]{1-{\left(\sum_{t=1}^{n}\varsigma_{t}{\left(1-{\left(\phi_{F_{t}}\right)}^{3}\right)}^{\sigma }\right)}^{\frac{1}{\sigma }}}\right)}^{3}-{\left(\sqrt[{3}]{{\left(\sum_{t=1}^{n}\varsigma_{t}{\left(\psi_{F_{t}}\right)}^{3\sigma }\right)}^{\frac{1}{\sigma }}}\right)}^{3}\\ \; & \;\;\leq {\left(\max_{1\leq t\leq n}\phi_{F_{t}}\right)}^{3}-{\left(\min_{1\leq t\leq n}\phi_{F_{t}}\right)}^{3}=SC\left(F^{+}\right) \end{array}$$
  and
  $$\begin{array}{cc} \; & SC\left(F\right)={\left(\phi_{F}\right)}^{3}-{\left(\psi_{F}\right)}^{3}={\left(\sqrt[{3}]{1-{\left(\sum_{t=1}^{n}\varsigma_{t}{\left(1-{\left(\phi_{F_{t}}\right)}^{3}\right)}^{\sigma }\right)}^{\frac{1}{\sigma }}}\right)}^{3}-{\left(\sqrt[{3}]{{\left(\sum_{t=1}^{n}\varsigma_{t}{\left(\psi_{F_{t}}\right)}^{3\sigma }\right)}^{\frac{1}{\sigma }}}\right)}^{3}\\ \; & \;\;\geq {\left(\min_{1\leq t\leq n}\phi_{F_{t}}\right)}^{3}-{\left(\max_{1\leq t\leq n}\phi_{F_{t}}\right)}^{3}=SC\left(F^{-}\right). \end{array}$$

 □

Accordingly, $F^{-}\leq FFSSWA\left(F_{1},F_{2},\cdots ,F_{n}\right)\leq F^{+}$ holds for all *t*.

**Property** **3** (Commutativity)**.** *Assume that ${\hat{F}}_{t}\left(t=1\left(1\right)n\right)$ is any permutation of $F_{t}\left(t=1\left(1\right)n\right)$. Then*

(16) $$\begin{array}{c} FFSSWA\left(F_{1},F_{2},\cdots ,F_{n}\right)=FFSSWA\left({\hat{F}}_{1},{\hat{F}}_{2},\cdots ,{\hat{F}}_{n}\right). \end{array}$$

**Proof.**  Based on the definition and theorem of the FFSSWA operator, we have

$$\begin{array}{cc} \; & FFSSWA\left(F_{1},F_{2},\cdots ,F_{n}\right)=\left(\sqrt[{3}]{1-{\left(\sum_{t=1}^{n}\varsigma_{t}{\left(1-{\left(\phi_{F_{t}}\right)}^{3}\right)}^{\sigma }\right)}^{\frac{1}{\sigma }}},\sqrt[{3}]{{\left(\sum_{t=1}^{n}\varsigma_{t}{\left(\psi_{F_{t}}\right)}^{3\sigma }\right)}^{\frac{1}{\sigma }}}\right)\\ \; & FFSSWA\left({\hat{F}}_{1},{\hat{F}}_{2},\cdots ,{\hat{F}}_{n}\right)=\left(\sqrt[{3}]{1-{\left(\sum_{t=1}^{n}{\hat{\varsigma }}_{t}{\left(1-{\left(\phi_{{\hat{F}}_{t}}\right)}^{3}\right)}^{\sigma }\right)}^{\frac{1}{\sigma }}},\sqrt[{3}]{{\left(\sum_{t=1}^{n}{\hat{\varsigma }}_{t}{\left(\psi_{{\hat{F}}_{t}}\right)}^{3\sigma }\right)}^{\frac{1}{\sigma }}}\right). \end{array}$$

Since $\left\{{\hat{F}}_{1},{\hat{F}}_{2},\cdots ,{\hat{F}}_{n}\right\}$ is any permutation of $\left\{F_{1},F_{2},\cdots ,F_{n}\right\}$. Then we have

$$\begin{array}{cc} \; & \sqrt[{3}]{1-{\left(\sum_{t=1}^{n}\varsigma_{t}{\left(1-{\left(\phi_{F_{t}}\right)}^{3}\right)}^{\sigma }\right)}^{\frac{1}{\sigma }}}=\sqrt[{3}]{1-{\left(\sum_{t=1}^{n}{\hat{\varsigma }}_{t}{\left(1-{\left(\phi_{{\hat{F}}_{t}}\right)}^{3}\right)}^{\sigma }\right)}^{\frac{1}{\sigma }}},\\ \; & \sqrt[{3}]{{\left(\sum_{t=1}^{n}\varsigma_{t}{\left(\phi_{F_{t}}\right)}^{3\sigma }\right)}^{\frac{1}{\sigma }}}=\sqrt[{3}]{{\left(\sum_{t=1}^{n}{\hat{\varsigma }}_{t}{\left(\phi_{{\hat{F}}_{t}}\right)}^{3\sigma }\right)}^{\frac{1}{\sigma }}}. \end{array}$$

Therefore, we have $FFSSWA\left(F_{1},F_{2},\cdots ,F_{n}\right)=FFSSWA\left({\hat{F}}_{1},{\hat{F}}_{2},\cdots ,{\hat{F}}_{n}\right)$.  □

When the parameter σ in an FFSSWA operator is taken as zero, then the FFSSWA operator will yield to the Fermatean fuzzy weighted averaging operator based on algebraic operations.

(17) $$\begin{array}{cc} FFSSWA_{\sigma =0}\left(F_{1},F_{2},\cdots ,F_{n}\right) & =\left(\sqrt[{3}]{1-\prod_{t=1}^{n}{\left(1-{\left(\phi_{F_{t}}\right)}^{3}\right)}^{\varsigma_{t}}},\prod_{t=1}^{n}{\left(\psi_{F_{t}}\right)}^{\varsigma_{t}}\right). \end{array}$$

**Definition** **10.** *Suppose $F_{t}=\left(\phi_{F_{t}},\psi_{F_{t}}\right)$ is a family of FFNs, and $\varsigma_{t}\left(t=1\left(1\right)n\right)$ is the weight of $\varsigma_{t}\in \left[0,1\right]$ with $\sum_{t=1}^{n}\varsigma_{t}=1$. The FFSSOWA operator is a mapping $\Theta^{n}\to \Theta$. If*

(18) $$\begin{array}{c} FFSSOWA\left(F_{1},F_{2},\cdots ,F_{n}\right)=\varsigma_{1}F_{\epsilon (1)}{⊕}_{SS}\varsigma_{2}F_{\epsilon (2)}{⊕}_{SS}\cdots {⊕}_{SS}\varsigma_{n}F_{\epsilon (n)}, \end{array}$$

*then the FFSSOWA is called a Fermatean fuzzy Schweizer–Sklar ordered weighted geometric operator, in which $\left(\epsilon (1),\epsilon (2),\cdots ,\epsilon (n)\right)$ is a permutation of $\left(1,2,\cdots ,n\right)$ within $F_{\epsilon (t-i)}\geq F_{\epsilon (t)}$ for t=2,3,⋯,n, and *Θ* signifies the set of FFNs.*

**Theorem** **3.** *Suppose $F_{t}=\left(\phi_{F_{t}},\psi_{F_{t}}\right)$ is a family of FFNs. Then the fusion value through utilizing the FFSSOWA operator is still an FFN and is represented as*

(19) $$\begin{array}{c} FFSSOWA\left(F_{1},F_{2},\cdots ,F_{n}\right)=\left(\begin{array}{c} \sqrt[{3}]{1-{\left(\sum_{t=1}^{n}\varsigma_{t}{\left(1-{\left(\phi_{F_{\epsilon (t)}}\right)}^{3}\right)}^{\sigma }\right)}^{\frac{1}{\sigma }}},\\ \sqrt[{3}]{{\left(\sum_{t=1}^{n}\varsigma_{t}{\left(\psi_{F_{\epsilon (t)}}\right)}^{3\sigma }\right)}^{\frac{1}{\sigma }}} \end{array}\right). \end{array}$$

### 4.3. Fermatean Fuzzy Schweizer–Sklar Weighted Geometric Operator

**Definition** **11.** *Suppose $F_{t}=\left(\phi_{F_{t}},\psi_{F_{t}}\right)$ is a family of FFNs and the FFSSWG operator is a mapping $\Theta^{n}\to \Theta$. If*

(20) $$\begin{array}{c} FFSSWG\left(F_{1},F_{2},\cdots ,F_{n}\right)={\left(F_{1}\right)}^{\varsigma_{1}}{⊗}_{SS}{\left(F_{2}\right)}^{\varsigma_{2}}{⊗}_{SS}\cdots {⊗}_{SS}{\left(F_{n}\right)}^{\varsigma_{n}}, \end{array}$$

*then the FFSSWG is called a Fermatean fuzzy Schweizer–Sklar weighted geometric operator, where *Θ* signifies the set of FFNs and $\varsigma_{t}\left(t=1\left(1\right)n\right)$ is the weight of $F_{t}$ with $\varsigma_{t}\in \left[0,1\right]$ with $\sum_{t=1}^{n}\varsigma_{t}=1$. Moreover, the FFSSWA operator will yield to the Fermatean fuzzy Schweizer–Sklar geometric operator when $\varpi_{t}={\left(\frac{1}{n},\frac{1}{n},\cdots ,\frac{1}{n}\right)}^{T}$.*

**Theorem** **4.** *Suppose $F_{t}=\left(\phi_{F_{t}},\psi_{F_{t}}\right)$ is a family of FFNs. Then the fusion value through employing the FFSSWG operator is still an FFN and represented as*

(21) $$\begin{array}{c} FFSSWG\left(F_{1},F_{2},\cdots ,F_{n}\right)=\left(\sqrt[{3}]{{\left(\sum_{t=1}^{n}\varsigma_{t}{\left(\phi_{F_{t}}\right)}^{3\sigma }\right)}^{\frac{1}{\sigma }}},\sqrt[{3}]{1-{\left(\sum_{t=1}^{n}\varsigma_{t}{\left(1-{\left(\psi_{F_{t}}\right)}^{3}\right)}^{\sigma }\right)}^{\frac{1}{\sigma }}}\right). \end{array}$$

The proof of Theorem 4 is similar to Theorem 1, so it is omitted here.

Similar to FFSSWG operator, the FFSSWG operator also possesses the idempotency, boundedness and commutativity. In addition, when the parameter σ in FFSSWG operator is taken zero, then the FFSSWG operator will yield to the Fermatean fuzzy weighted geometric operator based on algebraic operations.

(22) $$\begin{array}{cc} FFSSWG_{\sigma =0}\left(F_{1},F_{2},\cdots ,F_{n}\right) & =\left(\prod_{t=1}^{n}{\left(\phi_{F_{t}}\right)}^{\varsigma_{t}},\sqrt[{3}]{1-\prod_{t=1}^{n}{\left(1-{\left(\psi_{F_{t}}\right)}^{3}\right)}^{\varsigma_{t}}}\right). \end{array}$$

**Definition** **12.** *Suppose $F_{t}=\left(\phi_{F_{t}},\psi_{F_{t}}\right)$ is a family of FFNs, $\varsigma_{t}\left(t=1\left(1\right)n\right)$ is the weight of fusion-related with $\varsigma_{t}\in \left[0,1\right]$ with $\sum_{t=1}^{n}\varsigma_{t}=1$. FFSSOWG operator is a mapping from $\Theta^{n}$ to *Θ*. If*

(23) $$\begin{array}{c} FFSSOWG\left(F_{1},F_{2},\cdots ,F_{n}\right)={\left(F_{\epsilon (1)}\right)}^{\varsigma_{1}}{⊗}_{SS}{\left(F_{\epsilon (2)}\right)}^{\varsigma_{2}}{⊗}_{SS}\cdots {⊗}_{SS}{\left(F_{\epsilon (n)}\right)}^{\varsigma_{n}}, \end{array}$$

*then FFSSOWG is called a Fermatean fuzzy Schweizer–Sklar ordered weighted geometric operator, where ϵ(t)(t=1(1)n) is a permutation of $\left(1,2,\cdots ,n\right)$ within $F_{\epsilon (t-i)}\geq F_{\epsilon (t)}$ for t=2,3,⋯,n and *Θ* signifies the set of FFNs.*

**Theorem** **5.** *Suppose $F_{j}=\left(\zeta_{F_{j}},\eta_{F_{j}}\right)$ is a family of FFNs. Then the fusion value through utilizing the FFFOWG operator is still an FFN and represented as*

(24) $$\begin{array}{c} FFSSWG\left(F_{1},F_{2},\cdots ,F_{n}\right)=\left(\begin{array}{c} \sqrt[{3}]{{\left(\sum_{t=1}^{n}\varsigma_{t}{\left(\phi_{F_{\epsilon (t)}}\right)}^{3\sigma }\right)}^{\frac{1}{\sigma }}},\sqrt[{3}]{1-{\left(\sum_{t=1}^{n}\varsigma_{t}{\left(1-{\left(\psi_{F_{\epsilon (t)}}\right)}^{3}\right)}^{\sigma }\right)}^{\frac{1}{\sigma }}} \end{array}\right). \end{array}$$

The proof of Theorem 5 is similar to the Theorem 2, so it is omitted here.

## 5. An Integrated Fermatean Fuzzy CoCoSo Group-Decision Framework with Unknown Weight Information

In this section, we construct an integrated Fermatean fuzzy group decision framework on the basis of the best-worst method, entropy weight and CoCoSo to cope with MCGDM’s issue with unknown weight information. First, we define the MCGDM issue and obtain linguistic assessment information from experts based on their cognition and experience. Next, the weight information of experts is computed by a score function in which the weight information is provided by Fermatean fuzzy numbers from experts. Meanwhile, the fused assessment matrix is acquired through the proposed FFSSWA operator and expert weight. Further, criteria weights are ascertained from two aspects: subjective weight is determined by BWM based on entropy, and objective weight is identified using entropy weight. Lastly, the rank of the scheme is calculated based upon the improved CoCoSo method using the FFSSWA operator, the FFSSWG operator and the score function. A succinct Fermatean fuzzy group decision algorithm is also provided by summarizing the mentioned decision steps.

### 5.1. Problem Description

The classical MCGDM decision problem within a Fermatean fuzzy setting consists of the following fundamental notions. The scheme set denoted as $\Upsilon =\{\Upsilon_{s}|s=1\left(1\right)m\}$ is utilized as decision objects. The criterion set indicated as $C=\{C_{t}|t=1\left(1\right)n\}$ is viewed as assessment indexes with corresponding weights $\omega_{t}$ and $\omega_{t}\in \left[0,1\right]$, and $\sum_{t=1}^{n}\omega_{t}=1$. The assessment experts and corresponding weights are signified as $DE^{l}\left(l=1\left(1\right)L\right)$ and $\nu ={\left\{\nu_{1}\left(l=1\left(1\right)L\right)\right\}}^{T}$, with $\nu \in \left[0,1\right],\sum_{l=1}^{L}=1$. The evaluators $DE^{l}$ provide their assessment information for schemes $\Upsilon_{s}$ under the criteria $C_{t}$ by the form of FFN ${\bar{F_{st}}}^{l}=\left({\bar{\phi_{F_{st}}}}^{l},{\bar{\psi_{F_{st}}}}^{l}\right)$, where ${\bar{\phi_{F_{st}}}}^{l},{\bar{\psi_{F_{st}}}}^{l}\in \left[0,1\right]$ and $0\leq {\left({\bar{\phi_{F_{st}}}}^{l}\right)}^{3}+{\left({\bar{\psi_{F_{st}}}}^{l}\right)}^{3}\leq 1$. Hence, the decision matrices ${\bar{F}}^{l}={\left({\bar{F_{st}}}^{l}\right)}_{m\times n}$ are constructed by collecting all assessment information provided through experts for alternatives under different criteria, as shown below.

$$\begin{array}{c} {\bar{F}}^{l}={\left({\bar{F_{st}}}^{l}\right)}_{m\times n}=\left(\begin{array}{ccccc} {\bar{F_{11}}}^{l}=\left({\bar{\phi_{F_{11}}}}^{l},{\bar{\psi_{F_{11}}}}^{l}\right) & {\bar{F_{12}}}^{l}=\left({\bar{\phi_{F_{12}}}}^{l},{\bar{\psi_{F_{12}}}}^{l}\right) & \cdots & {\bar{F_{1n}}}^{l}=\left({\bar{\phi_{F_{1n}}}}^{l},{\bar{\psi_{F_{1n}}}}^{l}\right) & \\ {\bar{F_{21}}}^{l}=\left({\bar{\phi_{F_{21}}}}^{l},{\bar{\psi_{F_{21}}}}^{l}\right) & {\bar{F_{22}}}^{l}=\left({\bar{\phi_{F_{22}}}}^{l},{\bar{\psi_{F_{22}}}}^{l}\right) & \cdots & {\bar{F_{2n}}}^{l}=\left({\bar{\phi_{F_{2n}}}}^{l},{\bar{\psi_{F_{2n}}}}^{l}\right) & \\ \vdots & \vdots & \vdots & \vdots \\ {\bar{F_{m1}}}^{l}=\left({\bar{\phi_{F_{m1}}}}^{l},{\bar{\psi_{F_{m1}}}}^{l}\right) & {\bar{F_{m2}}}^{l}=\left({\bar{\phi_{F_{m2}}}}^{l},{\bar{\psi_{F_{m2}}}}^{l}\right) & \cdots & {\bar{F_{mn}}}^{l}=\left({\bar{\phi_{F_{mn}}}}^{l},{\bar{\psi_{F_{mn}}}}^{l}\right) & \end{array}\right) \end{array}$$

The goal of this research is to build an integrated group-decision methodology involving weight determination and scheme ranking based on decision matrices to resolve the MCGDM problem under Fermatan fuzzy surroundings.

### 5.2. The Steps of the Propounded Decision Approach

**Figure 1:**
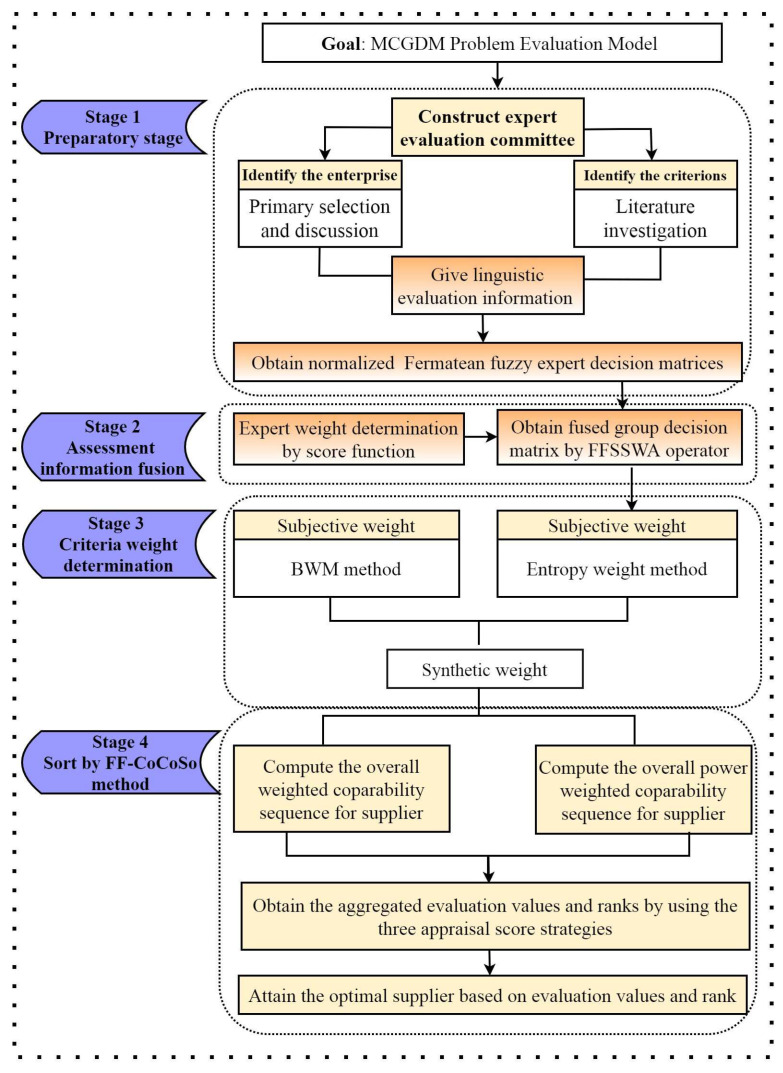
Fermatean fuzzy CoCoSo grou-decision framework.

This section shall expound on the decision steps of the propounded Fermatean fuzzy CoCoSo group-decision framework at length. The decision framework is divided into four phases: assessment information collection, assessment information fusion, determination of criterion weight and scheme sorting by CoCoSo. A visual flowchart of the proposed Fermatean fuzzy CoCoSo group-decision framework is provided and displayed as Figure 1.

#### 5.2.1. Obtain the Fermatean Fuzzy Assessment Information

Step 1: Achieving the linguistic assessment information.

In order to choose the optimal scheme from the scheme set, we first form an expert committee and invite them to provide their preferences for schemes in terms of the selected criteria. A mapping relation displayed in Table 1 from linguistic terms to FFNs is given for experts to more easily express their cognitive preference information.

Step 2: Getting normalized Fermatean fuzzy assessment information.

We first transform the linguistic assessment information of experts to Fermatean fuzzy assessment information with the help of Table 1. Next, we shift the negative criteria into positive criteria to avoid the effects of inconsistency brought by the type of criteria. Thus, the normalized Fermatean fuzzy assessment matrices $F^{l}={\left(F_{st}^{l}\right)}_{m\times n}$ are acquired by Equation (25).

(25) $$\begin{array}{c} F_{st}^{l}=\left(\phi_{F_{st}}^{l},\psi_{F_{st}}^{l}\right)=\left\{\begin{array}{cc} \left({\bar{\phi_{F_{st}}}}^{l},{\bar{\psi_{F_{st}}}}^{l}\right), & C_{t}\;\mathrm{is}\;\mathrm{benefit}\;\mathrm{criterion};\\ \left({\bar{\psi_{F_{st}}}}^{l},{\bar{\phi_{F_{st}}}}^{l}\right), & C_{t}\;\mathrm{is}\;\cos t\;\mathrm{criterion}. \end{array}\right. \end{array}$$

#### 5.2.2. Assessment Information Fusion

In order to ponder the group opinion of multiple decision experts, we assemble the individual assessment opinions into a single evaluation matrix to effectively develop the decision analysis. This includes expert weight calculation and assessment information integration.

Step 3: Evaluating the weight of decision experts.

Importance grades of experts in the course of decision analysis are often different since experts possess diverse cognition and experience on the objects. The presented decision framework considers the vagueness of experts and further expresses the importance of experts in the form of FFN. Suppose $F^{l}=\left(\phi_{F}^{l},\psi_{F}^{l}\right)$ is an FFN, then the weight $\nu_{l}$ of expert $DE_{l}$ is computed by Equation (26).

(26) $$\begin{array}{c} \nu_{l}=\frac{\frac{1}{2}\left(\left({\left(\phi_{F}^{l}\right)}^{3}-{\left(\psi_{F}^{l}\right)}^{3}-\ln\left(2-{\left(\phi_{F}^{l}\right)}^{3}-{\left(\psi_{F}^{l}\right)}^{3}\right)\right)+1\right)}{\sum_{l=1}^{L}\left(\frac{1}{2}\left(\left({\left(\phi_{F}^{l}\right)}^{3}-{\left(\psi_{F}^{l}\right)}^{3}-\ln\left(2-{\left(\phi_{F}^{l}\right)}^{3}-{\left(\psi_{F}^{l}\right)}^{3}\right)\right)+1\right)\right)},\;l=1,2,\cdots ,L. \end{array}$$

Step 4: Obtaining the synthesized assessment matrix.

On the basis of the expert matrices, the synthesize assessment matrix $F={\left(F_{st}\right)}_{m\times n}$ can be attained by the FFSSWA operator displayed in Equation (27).

(27) $$\begin{array}{c} F_{st}=FFSSWA\left(F_{st}^{1},F_{st}^{2},\cdots ,F_{st}^{L}\right)=\left(\begin{array}{c} \sqrt[{3}]{1-{\left(\sum_{t=1}^{n}\nu^{l}{\left(1-{\left(\phi_{F_{st}}^{1}\right)}^{3}\right)}^{\sigma }\right)}^{\frac{1}{\sigma }}},\\ \sqrt[{3}]{{\left(\sum_{t=1}^{n}\nu^{l}{\left(\psi_{F_{st}}^{1}\right)}^{3\sigma }\right)}^{\frac{1}{\sigma }}} \end{array}\right). \end{array}$$

#### 5.2.3. Computing the Criteria Weight Based on Combinative Method

In order to ascertain the importance of criteria during Fermatean fuzzy decision analysis, the current part presents a combinative weight determination model through merging BWM and entropy weight, which takes into account the influence of subjective preference and objective information simultaneously. It is worth noting that combinative weights are all built based upon the propounded Fermatean fuzzy entropy measure. The detailed computation process of combinative weights is illustrated as follows.

Step 5: Identifying the subjective weight $\omega_{t}^{sub}$ of criterion $C_{t}$ by using BWM based on entropy.

(1) Determine the best criterion $C_{B}$ and worst criterion $C_{W}$ from the criterion set based upon the knowledge and experience of the expert committee.
(2) To take into account the uncertainty of expert preferences, comparative vectors including best-to-others (BO) $BO=\left(B_{B1},B_{B2},\cdots ,B_{Bn}\right)$ and other-to-worst (OW) $OW={\left(W_{1W},W_{2W},\cdots ,W_{nW}\right)}^{T}$ are determined, in which $B_{Bt}$ and $W_{tW}$ are signified in the form of FFNs. The BO vector and OW vector denote the preference between the best criterion $C_{B}$ to other criteria $C_{t}$, and the preference between other criteria $C_{t}$ to the worst criterion $C_{W}$, respectively.
(3) Shift the BO and OW vectors to real number on the basis of the proposed Fermatean fuzzy entropy measure, as below:
  (28) $$\begin{array}{cc} \; & EBO=\left(E\left(B_{B1}\right),E\left(B_{B2}\right),\cdots ,E\left(B_{Bn}\right)\right), \end{array}$$
  (29) $$\begin{array}{cc} \; & EOW={\left(E\left(W_{1W}\right),E\left(W_{2W}\right),\cdots ,E\left(W_{nW}\right)\right)}^{T}, \end{array}$$
  where
  $$\begin{array}{cc} \; & E\left(B_{Bt}\right)=\frac{1}{2}\left[\frac{e^{-\left|{\left(\phi_{F}\right)}^{3}-{\left(\psi_{F}\right)}^{3}\right|}-e^{-1}}{1-e^{-1}}+\left(1-\left|{\left(\phi_{F}\right)}^{3}-{\left(\psi_{F}\right)}^{3}\right|\right)\right],\\ \; & E\left(W_{tW}\right)=\frac{1}{2}\left[\frac{e^{-\left|{\left(\phi_{F}\right)}^{3}-{\left(\psi_{F}\right)}^{3}\right|}-e^{-1}}{1-e^{-1}}+\left(1-\left|{\left(\phi_{F}\right)}^{3}-{\left(\psi_{F}\right)}^{3}\right|\right)\right] \end{array}$$
(4) Aiming at the EBO and EOW vectors, the multiplicative consistency relationships between Fermatean fuzzy entropy and criterion weight are indicated as:
  (30) $$\begin{array}{c} \overset{˘}{E}\left(B_{Bt}\right)=\frac{\omega_{B}^{sub}}{\omega_{B}^{sub}+\omega_{t}^{sub}},\;\overset{˘}{E}\left(W_{tW}\right)=\frac{\omega_{t}^{sub}}{\omega_{t}^{sub}+\omega_{W}^{sub}}, \end{array}$$
  in which $\overset{˘}{E}\left(B_{Bt}\right)=1-E\left(B_{Bt}\right)$, $\overset{˘}{E}\left(W_{tW}\right)=1-E\left(W_{tW}\right)$. Here, based on the information entropy, the smaller the entropy, the larger the entropy value. Hence, we utilize $\overset{˘}{E}\left(B_{Bt}\right)$ to replace $E\left(B_{Bt}\right)$ to ensure the consistency of comparison procedures.
(5) Further, we build the following model based on the proffered Fermatean fuzzy entropy measure.
  (31) $$\begin{array}{cc} \; & \min\chi \\ \; & s.t\;\left\{\begin{array}{c} \left|\frac{\omega_{B}^{sub}}{\omega_{B}^{sub}+\omega_{t}^{sub}}-\overset{˘}{E}\left(B_{Bt}\right)\right|\leq \chi \\ \left|\frac{\omega_{t}^{sub}}{\omega_{t}^{sub}+\omega_{W}^{sub}}-\overset{˘}{E}\left(W_{tW}\right)\right|\leq \chi \\ \sum_{t=1}^{n}\omega_{t}=1\\ \omega_{t}\geq 0. \end{array}\right. \end{array}$$

Based upon the results obtained in [63], the first model can be further shifted into

(32) $$\begin{array}{cc} \; & \min\chi_{1}\\ \; & s.t\;\left\{\begin{array}{c} \left|\omega_{B}^{sub}-\left(\omega_{B}^{sub}+\omega_{t}^{sub}\right)\times \overset{˘}{E}\left(B_{Bt}\right)\right|\leq \chi_{1}\\ \left|\omega_{t}^{sub}-\left(\omega_{t}^{sub}+\omega_{W}^{sub}\right)\times \overset{˘}{E}\left(W_{tW}\right)\right|\leq \chi_{1}\\ \sum_{t=1}^{n}\omega_{t}=1\\ \omega_{t}\geq 0. \end{array}\right. \end{array}$$

The model can be solved with the aid of LINGO software to further acquire the subjective weight $\omega_{t}^{sub}={\left(\omega_{1}^{sub},\omega_{2}^{sub},\cdots ,\omega_{n}^{sub}\right)}^{T}$.

Step 6: Identifying the objective weight $\omega_{t}^{obj}$ of criterion $C_{t}$ utilizing the entropy weight.

(1) Compute the entropy matrix $E={\left(E_{st}\right)}_{m\times n}$ based on the proposed Fermatean fuzzy entropy measure and the comprehensive matrix by Equation (33)
  (33) $$\begin{array}{c} E_{st}=\frac{1}{2n}\sum_{t=1}^{n}\left[\frac{e^{-\left|{\left(\phi_{F}\left(y_{t}\right)\right)}^{3}-{\left(\psi_{F}\left(y_{t}\right)\right)}^{3}\right|}-e^{-1}}{1-e^{-1}}+\left(1-\left|{\left(\phi_{F}\left(y_{t}\right)\right)}^{3}-{\left(\psi_{F}\left(y_{t}\right)\right)}^{3}\right|\right)\right] \end{array}$$
(2) Calculate the criterion weight by Equation (34).
  (34) $$\begin{array}{c} \omega_{t}^{obj}=\frac{1-\sum_{s=1}^{m}d_{st}}{\sum_{t=1}^{n}\left(1-\sum_{s=1}^{m}d_{st}\right)} \end{array}$$

Step 7: Identify the synthesized weight $\omega_{t}$ of criterion $C_{t}$ with the aid of Equation (35).

(35) $$\begin{array}{c} \omega_{t}=\frac{\sqrt{\omega_{t}^{obj}\omega_{t}^{sub}}}{\sum_{t=1}^{n}\sqrt{\omega_{t}^{obj}\omega_{t}^{sub}}},\;t=1,2,\cdots ,n. \end{array}$$

#### 5.2.4. Ranking by Improved Fermatean Fuzzy CoCoSo

Step 8: Compute the weighted sum measure by the FFSSWA operator.

(36) $$\begin{array}{c} {\tilde{P}}_{s}=FFSSWA\left(F_{s1},F_{s2},\cdots ,F_{sn}\right)=\left(\begin{array}{c} \sqrt[{3}]{1-{\left(\sum_{t=1}^{n}\omega_{t}{\left(1-{\left(\phi_{F_{st}}\right)}^{3}\right)}^{\sigma }\right)}^{\frac{1}{\sigma }}},\\ \sqrt[{3}]{{\left(\sum_{t=1}^{n}\omega_{t}{\left(\psi_{F_{st}}\right)}^{3\sigma }\right)}^{\frac{1}{\sigma }}} \end{array}\right) \end{array}$$

where ${\tilde{P}}_{s}=\left(\phi_{{\tilde{P}}_{s}},\psi_{{\tilde{P}}_{s}}\right)$ is the Fermatean fuzzy Schweizer–Sklar weighted average comparability sequence for scheme $\Upsilon_{s}$.

Step 9: Compute the weighted product measure by the FFSSWG operator.

(37) $$\begin{array}{c} {\tilde{Q}}_{s}=FFSSWA\left(F_{s1},F_{s2},\cdots ,F_{sn}\right)=\left(\begin{array}{c} \sqrt[{3}]{{\left(\sum_{t=1}^{n}\omega_{t}{\left(\phi_{F_{st}}\right)}^{3\sigma }\right)}^{\frac{1}{\sigma }}},\\ \sqrt[{3}]{1-{\left(\sum_{t=1}^{n}\omega_{t}{\left(1-{\left(\psi_{F_{st}}\right)}^{3}\right)}^{\sigma }\right)}^{\frac{1}{\sigma }}} \end{array}\right) \end{array}$$

where ${\tilde{Q}}_{s}=\left(\phi_{{\tilde{Q}}_{s}},\psi_{{\tilde{Q}}_{s}}\right)$ is the Fermatean fuzzy Schweizer–Sklar weighted geometric comparability sequence for scheme $\Upsilon_{s}$.

Step 10: Compute the appraisal score of the WSM and WPM.

(38) $$\begin{array}{cc} \; & S\left({\tilde{P}}_{s}\right)=\frac{1}{2}\left(\left({\left(\phi_{{\tilde{P}}_{s}}\right)}^{3}-{\left(\psi_{{\tilde{P}}_{s}}\right)}^{3}-\ln\left(2-{\left(\phi_{{\tilde{P}}_{s}}\right)}^{3}-{\left(\psi_{{\tilde{P}}_{s}}\right)}^{3}\right)\right)+1\right), \end{array}$$

(39) $$\begin{array}{cc} \; & S\left({\tilde{Q}}_{s}\right)=\frac{1}{2}\left(\left({\left(\phi_{{\tilde{Q}}_{s}}\right)}^{3}-{\left(\psi_{{\tilde{Q}}_{s}}\right)}^{3}-\ln\left(2-{\left(\phi_{{\tilde{Q}}_{s}}\right)}^{3}-{\left(\psi_{{\tilde{Q}}_{s}}\right)}^{3}\right)\right)+1\right). \end{array}$$

Step 11: Work out the relative importance of alternatives with the aid of three appraisal score strategies.

(i) Compute assessment score $G_{s}^{\left(1\right)}$ of scheme $\Upsilon_{s}$ through the arithmetic mean strategy displayed in Equation (40),
  (40) $$\begin{array}{c} G_{s}^{\left(1\right)}=\frac{S\left({\tilde{P}}_{s}\right)+S\left({\tilde{Q}}_{s}\right)}{\sum_{s=1}^{m}\left(S\left({\tilde{P}}_{s}\right)+S\left({\tilde{Q}}_{s}\right)\right)}. \end{array}$$
(ii) Compute assessment score $G_{s}^{\left(2\right)}$ of scheme $\Upsilon_{s}$ through the relative score strategy displayed in Equation (41),
  (41) $$\begin{array}{c} G_{s}^{\left(2\right)}=\frac{S\left({\tilde{P}}_{s}\right)}{\min_{1\leq s\leq m}\left\{S\left({\tilde{P}}_{s}\right)\right\}}+\frac{S\left({\tilde{Q}}_{s}\right)}{\min_{1\leq s\leq m}\left\{S\left({\tilde{Q}}_{s}\right)\right\}}. \end{array}$$
(iii) Compute assessment score $G_{s}^{\left(3\right)}$ of scheme $\Upsilon_{s}$ through the balanced compromise strategy displayed in Equation (42),
  (42) $$\begin{array}{c} G_{s}^{\left(3\right)}=\frac{\varrho S\left({\tilde{P}}_{s}\right)+\left(1-\varrho \right)S\left({\tilde{Q}}_{s}\right)}{\varrho \max_{1\leq s\leq m}\left\{S\left({\tilde{P}}_{s}\right)\right\}+\left(1-\varrho \right)\max_{1\leq s\leq m}\left\{S\left({\tilde{Q}}_{s}\right)\right\}}, \end{array}$$
  where ϱ(ϱ∈[0,1]) stands for the balancing coefficient.

Step 12: The classical COCOSO method only obtains the final decision scheme ranking by synthesizing the numerical results of the above three strategies, but ignores the ranking results of the three strategies. Based upon this defect, Wen et al. [48] constructed an innovative aggregation formulation to reasonably integrate the numerical results and rank the mentioned strategies. In this paper, we fuse the three aggregation strategies $G_{s}^{\left(y\right)}\left(y=1,2,3\right)$ and acquire the ultimate sorting of scheme $\Upsilon_{s}$.

(43) $$\begin{array}{c} ℜ\left(\Upsilon_{s}\right)=\sum_{y=1}^{3}\sqrt{\frac{1}{2}\left({\left(\frac{G_{s}^{\left(y\right)}}{\max_{1\leq s\leq m}\left\{G_{s}^{\left(y\right)}\right\}}\right)}^{2}+{\left(\frac{m-{\tilde{R}}^{\left(y\right)}}{m}\right)}^{2}\right)}, \end{array}$$

where $G_{s}^{\left(y\right)}$ denotes the strategy values of the *s*th strategy to supplier $\Upsilon_{s}$, and ${\tilde{R}}^{\left(y\right)}$ signifies the rank of supplier $\Upsilon_{s}$ under the strategy values of $G_{s}^{\left(y\right)}$, and *y* indicates the number of fusion strategies.

Step 13: End.

## 6. Empirical Study

In the current section, an empirical method for supplier assessment is employed to validate the feasibility and practicability of the proposed Fermatean fuzzy CoCoSo group-decision framework. To begin with, we illustrate the example’s background and use the presented FF-CoCoSo group-decision algorithm to deal with the supplier problem. Then we analyze the sensibility and robustness of the FF-CoCoSo group-decision algorithm with the aid of paramater analysis and weight during the decision analysis. Further, we expound several merits and significant characteristics of the advanced FF-CoCoSo group-decision algorithm in dealing with actual vague decisions.

### 6.1. Case Background

Selecting appropriate green suppliers is one of the important measures for enterprises to enhance their core competitiveness. Friendly cooperation between enterprises and suppliers does not only ensure the safety of commodity transportation, but also continuously improves the economics of enterprises. The advent of new energy vehicles not only reduces the air pollution caused by traditional fuel vehicles, but also further improves the combustion efficiency of automobile engines. ABC company is a large automobile company that produces new energy vehicles and related accessories and facilities. After the transformation of the company, the company will re-screen a number of green suppliers to provide services for the company. The transportation department of the enterprise selected six suppliers $\left\{\Upsilon_{s}|s=1,2,\cdots ,6\right\}$ through qualification review, enterprise credit review and other links to enter the expert selection stage. Four experts $\left\{E^{\left(l\right)}|l=1,2,3,4\right\}$ selected the best supplier by investigating the enterprise’s products and combining their own knowledge and experience to determine five criteria $\left\{C_{t}|t=1,2,\cdots ,5\right\}$. The illustrations of the above five criteria are displayed in Table 2. The core goal of the company is to acquire priority order and choose the best one of the six suppliers with the aid of four experts.

### 6.2. Decision Analysis

Stage 1: Obtain the Fermatean fuzzy assessment information.

Based on the introduction of the practical case, we use the presented FF-COCOSO group-decision approach to select the optimal supplier. The assessment experts give their assessment opinion for the considered supplier with respect to different criteria using the provided linguistic assessment terms; this assessment information is gathered in Table 3. Then, the Fermatean fuzzy assessment information is shown in Table 4, which is obtained through transformation of the data in Table 1.

Stage 2: Assessment information fusion.

Considering that different types of evaluation criteria will lead to unreasonable decision-making results, normalized Fermatean fuzzy assessment information is obtained according to Equation (25), and the results are displayed in Table 5.

Considering the fuzziness of experts’ cognition in decision analysis, experts provide their importance grades with the help of Fermatean fuzzy numbers, namely, $E^{\left(1\right)}=\left(0.90,0.30\right)$, $E^{\left(2\right)}=\left(0.80,0.40\right)$, $E^{\left(3\right)}=\left(0.70,0.50\right)$, $E^{\left(4\right)}=\left(0.75,0.45\right)$. Afterwards, the importance of each evaluation expert can be calculated based on the Fermatean fuzzy information and Equation (26). The result of expert weight $\nu_{l}$ is shown below:

$$\begin{array}{c} \nu_{l}=0.3447,\;\nu_{2}=0.2543,\;\nu_{3}=0.1839;\;\nu_{4}=0.2170. \end{array}$$

After that, the Fermatean fuzzy comprehensive assessment information of suppliers is acquired by aggregating the assessment information of diverse experts with the assistance of weight information and the proposed FFSSWA operators. The outcome is shown in Table 6.

Stage 3: Computing the criteria weight based on combinative method.

To compute the importance grade of the considered assessment criteria, we utilize the Fermatean fuzzy BWM and entropy weight to evaluate the subjective weight and objective weight, respectively, of assessment criteria. Then we further work out the combinative weight of criteria through integrating the ideal between the subjective and objective weights.

First, the subjective weights of criteria are determined by the Fermatean fuzzy BWM based on entropy. The experts select $C_{1}$ and $C_{4}$ as the best and worst criteria after discussion and negotiation. At the same time, the BO and OW vectors provided by experts are displayed as follows:

$$\begin{array}{cc} \; & FFBO=\left(\left(0.50,0.50\right),\left(0.85,0.25\right),\left(0.90,0.15\right),\left(0.95,0.25\right),\left(0.80,0.50\right)\right),\\ \; & FFOW={\left(\left(0.95,0.25\right),\left(0.80,0.15\right),\left(0.85,0.35\right),\left(0.50,0.50\right),\left(0.90,0.20\right)\right)}^{T}. \end{array}$$

Then, we compute the entropy value of FFBO and FFOW vectors via the proposed Fermatean fuzzy entropy measure displayed in Equation (4), namely, EBO=(1.0000,0.3445,0.2291,0.1290,0.5527), $EOW=\left(0.1290,0.4303,0.3702,1.0000,0.2331\right)$. Based on the results of EBO and EOW, we apply them to the BWM model and attain the following:

$$\begin{array}{cc} \; & \min\chi_{1}\\ \; & s.t\;\left\{\begin{array}{c} \left|\omega_{B}^{sub}-\left(\omega_{B}^{sub}+\omega_{1}^{sub}\right)\times 1.0000\right|\leq \chi_{1}\\ \left|\omega_{B}^{sub}-\left(\omega_{B}^{sub}+\omega_{2}^{sub}\right)\times 0.3445\right|\leq \chi_{1}\\ \left|\omega_{B}^{sub}-\left(\omega_{B}^{sub}+\omega_{3}^{sub}\right)\times 0.2291\right|\leq \chi_{1}\\ \left|\omega_{B}^{sub}-\left(\omega_{B}^{sub}+\omega_{4}^{sub}\right)\times 0.1290\right|\leq \chi_{1}\\ \left|\omega_{B}^{sub}-\left(\omega_{B}^{sub}+\omega_{5}^{sub}\right)\times 0.5527\right|\leq \chi_{1}\\ \left|\omega_{2}^{sub}-\left(\omega_{2}^{sub}+\omega_{W}^{sub}\right)\times 0.4303\right|\leq \chi_{1}\\ \left|\omega_{3}^{sub}-\left(\omega_{3}^{sub}+\omega_{W}^{sub}\right)\times 0.3702\right|\leq \chi_{1}\\ \left|\omega_{5}^{sub}-\left(\omega_{5}^{sub}+\omega_{W}^{sub}\right)\times 0.2331\right|\leq \chi_{1}\\ \sum_{t=1}^{n}\omega_{t}=1,\omega_{t}\geq 0\left(t=1,2,\cdots ,5\right). \end{array}\right. \end{array}$$

with this model, we can attain the subjective weight of criteria shown as $\omega_{1}^{sub}=0.0420,$$\omega_{2}^{sub}=0.2019,\omega_{3}^{sub}=0.2812,\omega_{4}^{sub}=0.3649,\omega_{5}^{sub}=0.1100.$ The consistency index $\chi_{1}=0.0420$, which shows high consistency is maintained during the weight determination process.

Next, the objective weights of criteria are computed based on the Fermatean fuzzy entropy weight using Equation (34); the outcomes are shown as below:

$$\begin{array}{c} \omega_{1}^{obj}=0.1493,\;\omega_{2}^{obj}=0.2611,\;\omega_{3}^{obj}=0.1750,\;\omega_{4}^{obj}=0.2147,\;\omega_{5}^{obj}=0.1999. \end{array}$$

Finally, the comprehensive weights of criteria are ascertained by Equation (35); the results are as follows:

$$\begin{array}{c} \omega_{1}=0.0826,\;\omega_{2}=0.2395,\;\omega_{3}=0.2313,\;\omega_{4}=0.2919,\;\omega_{5}=0.1547. \end{array}$$

Stage 4: Ranking by improved Fermatean fuzzy CoCoSo.

After obtaining the comprehensive weight of criteria by combining the BWM and entropy, we ascertain the rank order of suppliers by using Fermatean fuzzy CoCoSo, which is improved by the propounded FFSSWA and FFSSWG operators and score function. The computation of sum measure ${\tilde{P}}_{s}$ is by the FFSSWA operator and product measures ${\tilde{Q}}_{s}$ is by the FFSSWG operator, and scores are displayed in Table 7.

On the basis of the sum measure and product measure of suppliers, we first compute the assessment scores of suppliers by three strategies $G_{s}^{\left(1\right)},G_{s}^{\left(2\right)}$ and $G_{s}^{\left(3\right)}$. We acquire the rank order of suppliers by Equation (43) and list the corresponding computation outcomes in Table 8. From Table 8, the order of the selected green suppliers is $\Upsilon_{6}≻\Upsilon_{4}≻\Upsilon_{3}≻\Upsilon_{1}≻\Upsilon_{5}≻\Upsilon_{2}$. In a word, the optimal green supplier is $\Upsilon_{6}$.

### 6.3. Sensibility Analysis

Since parameters and weights play an important role in Fermatean fuzzy decision analysis, this subsection will perform a sensitivity discussion with respect to the different parameters and divers kinds of weights involved in the proposed Fermatean fuzzy MCGDM method, including the following two topics: (1) the influence of parameter σ in the presented FFSSWA and FFSSWG operators and parameter ϱ in the Fermatean fuzzy CoCoSo method on the final ranks of suppliers; (2) the fluctuation of the decision outcome attained through taking different types of weights in the process of supplier selection.

*Parameter change analysis.* We analyze the impact of parameters σ and ϱ for the final supplier ranking. The parameter σ exists in the FFSSWA operator and FFSSWG operator, which means it may influence expert information fusion and supplier assessment information integration. We take the value of σ as {−1,−2,−5,−13,−20,−50,−100} and further obtain the corresponding decision outcomes and ranks of suppliers, which are shown as Table 9 and Table 10. The results shown that although the comprehensive assessment values of different suppliers is different, the final rank is $\Upsilon_{6}≻\Upsilon_{4}≻\Upsilon_{1}≻\Upsilon_{3}≻\Upsilon_{5}≻\Upsilon_{2}$ or $\Upsilon_{6}≻\Upsilon_{4}≻\Upsilon_{3}≻\Upsilon_{1}≻\Upsilon_{5}≻\Upsilon_{2}$. However, the most satisfactory is always the sixth supplier, which means the proposed Fermatean fuzzy decision approach in this paper is stable with respect to diverse values of σ. Next, parameter ϱ can be regarded as a balance coefficient to regulate the proportion of WSM and WPM in the third fusion strategy. Based on the range of balance coefficient ϱ, we take the value of ϱ from 0.1 to 1 and determine the corresponding assessment values and ranks of suppliers. The results show that changing ϱ does not substantially change the ranking of suppliers, which shows that the proposed method is stable for different values of ϱ.

*Weight change analysis.* The weights of diverse supplier criteria are essential for experts to make a reasonable and effective selection from a group of suppliers. According to classifications of weight determination, we analyze the influence of different weight types on the final supplier ranking. Because the presented approach selects the optimal supplier based on integrated criteria weight to comprehensively factor objectivity and subjectivity into the decision analysis, we utilize four kinds of criteria weight, including objective weight, subjective weight, integrated weight and equal weight to recompute the mentioned supplier selection problem; the decision outcomes are displayed in Table 11. According to the acquired outcomes, we find that diverse criteria weights make minor changes to the outcome. The ranks of suppliers obtained by subjective weight and combined weight are the same, but they are different from the rank order attained by objective weight, which means that objective weight is non-ignorable for supplier ranking in this paper. Actually, the objective weight determined by practical decision data is very important for experts to develop a rational decision analysis for different complex decision problems.

### 6.4. Comparative Analysis

In this part, a comparison analysis with several previous Fermatean fuzzy decision approaches is implemented to further expound the efficiency and practicability of the proposed methodology. Four decision methodologies—Fermatean fuzzy TOPSIS (FF-TOPSIS), Fermatean fuzzy WASPAS (FF-WASPAS), Fermatean fuzzy WPM (FF-WPM), Fermatean fuzzy VIKOR (FF-VIKOR), Fermatean fuzzy ARAS (FF-ARAS) and Fermatean fuzzy SAW (FF-SAW)—are employed to sort the suppliers in this research. Among them, diverse decision techniques are extended to Fermatean fuzzy settings based on the distance, score and aggregation. In order to guarantee the rationality of the comparison process, we utilize the combined weight determined in this study to solve the above supplier problem based on the four listed methods. The rankings of suppliers deduced by the aforementioned methods are displayed in Table 12.

As can be observed from Table 12, the sorting outcomes of suppliers through employing the proposed approach is basically the same with the extant research FF-ARAS and FF-SAW methods. Besides, although these approaches achieve two rankings of suppliers, the most satisfactory suppliers as determined by FF-TOPSIS, FF-WASPAS, FF-WPM, FF-VIKOR, FF-ARAS, FF-SAW and the presented approaches is $\Upsilon_{6}$. Accordingly, the validity and applicability of the designed FF-CoCoSo approach is validated. Furthermore, we further analyze the differences and outcomes of the presented method with the mentioned approaches from the aspects of weight determination and ranking method.

*From the point of view of weight evaluation*. Previous approaches assume in advance that the weights of criteria are given by experts according to their subjective experience, knowledge background and cognitive ability, which often puts too much reliance on the professional knowledge of experts and leads to irrational decisions. By comparison, the propounded approach not only considers the subjective weight computed by an improved BWM, but it also factors the objective weight identified by entropy, which further enhances the practicality of the proposed approach in dealing with actual complicated decisions. Besides, the weight analysis in the section of sensitivity also proves that the different kinds of weight will affect the final ranks of suppliers. Therefore, the combined weight of criteria determined in our method is reasonable and advantageous for uncertain decision analysis.

*From the aspect of decision methods*. FF-TOPSIS and FF-VIKOR are based on distance between the ideal and negative ideal solutions and the assessment value to rank the suppliers. FF-WASPAS, FF-WPM, FF-ARAS and FF-SAW rank suppliers according to utility. However, these Fermatean fuzzy decision approaches have the following two defects: (1) they are all based on one or two integration strategies that fail to consider the influence of a balanced compromise strategy on the decision result; (2) they ignore the ranks of different integration strategies when the rank of a supplier is computed to be greater than or equal to two strategies. The proposed Fermatean fuzzy decision method deals with these two disadvantages efficaciously and thus obtains more rational and consistent decision outcomes.

With the assistance of the aforementioned comparison discussion, we further extract the significant features of these compared approaches according to the main characteristics of MCGDM. In Table 13, we contrast the presented method with previous Fermatean fuzzy decision methodologies from the aspects of weight determination, information fusion and ranking method, which further highlights the unique advantages of the method developed in this study. In view of the above-mentioned analysis and discussions, several merits of the proffered FF-CoCoSo group-decision approach are summarized as follows:

♣ The presented approach under Fermatean fuzzy setting can efficaciously attain the optimal scheme in an uncertain environment with completely unknown weight information of experts and criteria.
♣ The presented FF-CoCoSo group-decision method is improved based on the FFSSWA and FFSSWG operators to make the overall decision procedure more flexible through adjustable parameters.
♣ The identification of supplier criteria weights takes the subjective preferences and actual decision data into consideration simultaneously, which further strengthens the reliability and credibility of the decision outcomes.
♣ The final rank of suppliers is ascertained with the aid of an improved CoCoSo, which not only considers the numerical result of multiple strategies but also considers their rank outcomes. Accordingly, the ultimate rank result of suppliers is more credible and robust than some extant methods.

## 7. Results, Discussion and Conclusions

### 7.1. Results

In this research, we construct an multistage group decision approach with Fermatean fuzzy information to evaluate and select the most appropriate green supplier. Firstly, the numerical example to select a green supplier validates that the proffered method has high feasibility to support expert evaluation of green suppliers. Next, we conduct the sensibility discussion for the parameters in the process of information aggregation and different criteria weights. The results exhibit that the supplier ranking results obtained by the proposed method are stable no matter how the parameters change. However, the criteria weight from different perspectives makes the supplier ranking change slightly, but the optimal selection is the same. Finally, we perform comparative analysis with other priori Fermatean fuzzy decision methodologies to test the effectiveness of the presented method. The comparative results demonstrate that the constructed method is efficacious for experts to select the most satisfactory supplier. Meanwhile, characteristic comparison outcomes further highlight the significant advantage of the introduced method to rank green suppliers under complex, uncertain circumstances.

### 7.2. Discussion

As an effective extension of intuitionistic FS and Pythagorean FS, FFS does not only possess a stronger ability to express ambiguous information but also provides freer space for decision experts to express their preferences. In order to fully realize its advantages in depicting uncertain information and in considering the objectivity and subjectivity of decision processes, an integrated MCGDM decision methodology is presented to deal with practical decision problems with unknown weight information. In order to construct the MCGDM approach, some new Fermatean fuzzy Schweizer–Sklar operators are suggested to fuse Fermatean fuzzy information to improve the BWM, and a novel entropy measure is defined to improve the entropy weight method. Then, an improved CoCoSo method is attained based on the propounded Fermatean fuzzy Schweizer–Sklar operators and score function. The proposed Fermatean fuzzy group decision approach determines the rank of green suppliers by combining numerical results and order outcomes of the three fusion strategies, which further strengthen the rationality and reliability of the final green supplier ranking. The combination of BWM-Entropy–CoCoSo method is based on the proposed aggregation operators and entropy measure. The combined weight is attained by using BWM and entropy weight based on proposed Fermatean fuzzy entropy, which further improves the reliability of the criteria weight information. The improved CoCoSo method using the presented operators further enhances the flexibility and robustness of the final decision. Therefore, we merge the BWM-Entropy and CoCoSo method to construct a group decision framework to strengthen the rationality and feasibility of the decision outcomes.

### 7.3. Conclusions

In this research, a comprehensive group decision methodology is designed on the basis of synthetic weight calculation and a CoCoSo algorithm to rank green suppliers with Fermatean fuzzy information. Specifically, the Fermatean fuzzy Schweizer–Sklar operational laws are based on the Schweizer–Sklar t-norm and t-conorm, and then some Fermatean fuzzy Schweizer–Sklar aggregation operators are put forward based on the proposed operations. Secondly, a novel Fermatean fuzzy entropy measure is advanced to measure the fuzziness of FFS. Furthermore, the comprehensive weights of criteria are ascertained based on the improved BWM and entropy weight. Thirdly, an innovative Fermatean fuzzy MCGDM method is presented based on BWM-Entropy and CoCoSo to settle complicated decision issues; this model can overcome the defects of the extant methods that only consider the weight information of a single aspect. Ultimately, an actual problem about green supplier selection is applied to illustrate the effectiveness and feasibility of our propounded approach. Moreover, comparison between our proposed method and previous Fermatean fuzzy decision approaches is implemented to validate the validity and prominent superiorities of the designed group-decision method. The outcomes show that the proffered method possesses a certain availability and unique advantages.

Nevertheless, the proposed method also has some limitations: (a) it ignores the objective weight information of experts; (b) it fails to consider the consistency of experts during expert information fusion; (c) it assumes that the experts in the decision process are completely rational.

In view of the defects in the proffered method, future works will develop the research of uncertain decision methods and applications from the following aspects: (1) propose several novel Fermatean fuzzy Schweizer–Sklar operators by combining power average operators, Muirhead mean and partitioned Hamy Mean Operators; (2) explore and discuss some novel information measures such as dissimilar measures, knowledge measures and divergence measures to support Fermatean fuzzy decision analysis; (3) utilize the proposed group-decision method to address some realistic decision methods such as sustainable supply chain [64], emergency scheme assessment [65] and solid waste management [66]; (4) construct a large-scale group-decision-making model based on consensus reaching [67,68,69].

## Figures and Tables

**Table 1:** Linguistic terms for experts to choose green suppliers.

Linguistic term	Abbreviation	Fermatean Fuzzy Element
Very Very Low	VVL	(0.25, 0.95)
Very Low	VL	(0.30, 0.90)
Low	L	(0.35, 0.85)
Middle Low	ML	(0.40, 0.80)
Below Middle	BM	(0.50, 0.70)
Middle	M	(0.60, 0.60)
Above Middle	AM	(0.70, 0.50)
Middle High	MH	(0.80, 0.40)
High	H	(0.85, 0.35)
Very High	VH	(0.90, 0.30)
Very Very High	VVH	(0.95, 0.25)

**Table 2:** Depictions of the criteria for green supplier selection.

Criteria	Description	Type	References
Quality ($C_{1}$)	Quality is the characteristic that the supplier’s products meet the specified and potential needs, which is mainly reflected in the product qualification rate, quality stability, product repair and return rate and product cleanliness.	Benefit	[4,7,8,11,12,13,14]
Cost ($C_{2}$)	Cost is the main cost involved in the supplier’s service process, including service cost and transportation cost.	Cost	[3,4,7,8,11,12,13,14]
Service level ($C_{3}$)	This refers to the ability of suppliers to provide various services for the whole supply chain during delivery, which is mainly reflected in on-time arrival rate, flexibility of delivery ability, maintenance service ability and service attitude.	Benefit	[4,8,11,12,14]
Production capacity ($C_{4}$)	This is mainly reflected in the product production scale, the operation status of production equipment and the flexibility in the production process.	Benefit	[4,7,11,12,13]
Technical level ($C_{5}$)	This is mainly reflected in the ability for product innovation, the technical level of production equipment and the level of product design.	Benefit	[3,4,7,11,14]

**Table 3:** Preferences for selection of green supplier provided by experts using the linguistic terms.

Expert	Alternative	$C_{1}$	$C_{2}$	$C_{3}$	$C_{4}$	$C_{5}$
*E* ^(1)^	$\Upsilon_{1}$	VH	L	VH	VH	H
	$\Upsilon_{2}$	VH	VL	MH	MH	MH
	$\Upsilon_{3}$	MH	BM	VVH	H	MH
	$\Upsilon_{4}$	VVH	ML	M	AM	VH
	$\Upsilon_{5}$	MH	L	VH	VH	VH
	$\Upsilon_{6}$	VH	VVL	H	VH	VVH
*E* ^(2)^	$\Upsilon_{1}$	VH	ML	AM	H	M
	$\Upsilon_{2}$	VVH	L	M	MH	AM
	$\Upsilon_{3}$	MH	BM	VH	VH	M
	$\Upsilon_{4}$	AM	M	VH	AM	VVH
	$\Upsilon_{5}$	VH	L	MH	H	VH
	$\Upsilon_{6}$	VH	VL	VVH	VH	VH
*E* ^(3)^	$\Upsilon_{1}$	MH	VL	MH	H	AM
	$\Upsilon_{2}$	VH	BM	H	AM	M
	$\Upsilon_{3}$	MH	BM	M	VH	MH
	$\Upsilon_{4}$	VVH	L	VVH	MH	VH
	$\Upsilon_{5}$	VH	M	VH	VH	H
	$\Upsilon_{6}$	VH	VL	VH	H	VVH
*E* ^(4)^	$\Upsilon_{1}$	M	BM	VH	H	VVH
	$\Upsilon_{2}$	VH	L	AM	MH	MH
	$\Upsilon_{3}$	H	VL	VH	VH	VH
	$\Upsilon_{4}$	VH	VL	VH	VVH	M
	$\Upsilon_{5}$	M	BM	H	MH	H
	$\Upsilon_{6}$	VVH	VVL	VVH	H	VH

**Table 4:** Preferences for selection of green supplier provided by experts using Fermatean fuzzy information.

Expert	Alternative	$C_{1}$	$C_{2}$	$C_{3}$	$C_{4}$	$C_{5}$
*E* ^(1)^	$\Upsilon_{1}$	(0.90, 0.30)	(0.35, 0.85)	(0.90, 0.30)	(0.90, 0.30)	(0.85, 0.35)
	$\Upsilon_{2}$	(0.90, 0.30)	(0.30, 0.90)	(0.80, 0.40)	(0.80, 0.40)	(0.80, 0.40)
	$\Upsilon_{3}$	(0.80, 0.40)	(0.50, 0.70)	(0.95, 0.25)	(0.85, 0.35)	(0.80, 0.40)
	$\Upsilon_{4}$	(0.95, 0.25)	(0.40, 0.80)	(0.60, 0.60)	(0.70, 0.50)	(0.90, 0.30)
	$\Upsilon_{5}$	(0.80, 0.40)	(0.35, 0.85)	(0.90, 0.30)	(0.90, 0.30)	(0.90, 0.30)
	$\Upsilon_{6}$	(0.90, 0.30)	(0.25, 0.95)	(0.85, 0.35)	(0.90, 0.30)	(0.95, 0.25)
*E* ^(2)^	$\Upsilon_{1}$	(0.90, 0.30)	(0.40, 0.80)	(0.70, 0.50)	(0.85, 0.35)	(0.60, 0.60)
	$\Upsilon_{2}$	(0.95, 0.25)	(0.35, 0.85)	(0.60, 0.60)	(0.80, 0.40)	(0.70, 0.50)
	$\Upsilon_{3}$	(0.80, 0.40)	(0.50, 0.70)	(0.90, 0.30)	(0.90, 0.30)	(0.60, 0.60)
	$\Upsilon_{4}$	(0.70, 0.50)	(0.60, 0.60)	(0.90, 0.30)	(0.70, 0.50)	(0.95, 0.25)
	$\Upsilon_{5}$	(0.90, 0.30)	(0.35, 0.85)	(0.80, 0.40)	(0.85, 0.35)	(0.90, 0.30)
	$\Upsilon_{6}$	(0.90, 0.30)	(0.30, 0.90)	(0.95, 0.25)	(0.90, 0.30)	(0.90, 0.30)
*E* ^(3)^	$\Upsilon_{1}$	(0.80, 0.40)	(0.30, 0.90)	(0.80, 0.40)	(0.85, 0.35)	(0.70, 0.50)
	$\Upsilon_{2}$	(0.90, 0.30)	(0.50, 0.70)	(0.85, 0.35)	(0.70, 0.50)	(0.60, 0.60)
	$\Upsilon_{3}$	(0.80, 0.40)	(0.50, 0.70)	(0.60, 0.60)	(0.90, 0.30)	(0.80, 0.40)
	$\Upsilon_{4}$	(0.95, 0.25)	(0.35, 0.85)	(0.95, 0.25)	(0.80, 0.40)	(0.90, 0.30)
	$\Upsilon_{5}$	(0.90, 0.30)	(0.60, 0.60)	(0.90, 0.30)	(0.90, 0.30)	(0.85, 0.35)
	$\Upsilon_{6}$	(0.90, 0.30)	(0.30, 0.90)	(0.90, 0.30)	(0.85, 0.35)	(0.95, 0.25)
*E* ^(4)^	$\Upsilon_{1}$	(0.60, 0.60)	(0.50, 0.70)	(0.90, 0.30)	(0.85, 0.35)	(0.95, 0.25)
	$\Upsilon_{2}$	(0.90, 0.30)	(0.35, 0.85)	(0.70, 0.50)	(0.80, 0.40)	(0.80, 0.40)
	$\Upsilon_{3}$	(0.85, 0.35)	(0.30, 0.90)	(0.90, 0.30)	(0.90, 0.30)	(0.90, 0.30)
	$\Upsilon_{4}$	(0.90, 0.30)	(0.30, 0.90)	(0.90, 0.30)	(0.95, 0.25)	(0.60, 0.60)
	$\Upsilon_{5}$	(0.60, 0.60)	(0.50, 0.70)	(0.85, 0.35)	(0.80, 0.40)	(0.85, 0.35)
	$\Upsilon_{6}$	(0.95, 0.25)	(0.25, 0.95)	(0.95, 0.25)	(0.85, 0.35)	(0.90, 0.30)

**Table 5:** The normalized Fermatean fuzzy assessment information for selection of green supplier.

Expert	Alternative	$C_{1}$	$C_{2}$	$C_{3}$	$C_{4}$	$C_{5}$
*E* ^(1)^	$\Upsilon_{1}$	(0.90, 0.30)	(0.85, 0.35)	(0.90, 0.30)	(0.90, 0.30)	(0.85, 0.35)
	$\Upsilon_{2}$	(0.90, 0.30)	(0.90, 0.30)	(0.80, 0.40)	(0.80, 0.40)	(0.80, 0.40)
	$\Upsilon_{3}$	(0.80, 0.40)	(0.70, 0.50)	(0.95, 0.25)	(0.85, 0.35)	(0.80, 0.40)
	$\Upsilon_{4}$	(0.95, 0.25)	(0.80, 0.40)	(0.60, 0.60)	(0.70, 0.50)	(0.90, 0.30)
	$\Upsilon_{5}$	(0.80, 0.40)	(0.85, 0.35)	(0.90, 0.30)	(0.90, 0.30)	(0.90, 0.30)
	$\Upsilon_{6}$	(0.90, 0.30)	(0.95, 0.25)	(0.85, 0.35)	(0.90, 0.30)	(0.95, 0.25)
*E* ^(2)^	$\Upsilon_{1}$	(0.90, 0.30)	(0.80, 0.40)	(0.70, 0.50)	(0.85, 0.35)	(0.60, 0.60)
	$\Upsilon_{2}$	(0.95, 0.25)	(0.85, 0.35)	(0.60, 0.60)	(0.80, 0.40)	(0.70, 0.50)
	$\Upsilon_{3}$	(0.80, 0.40)	(0.70, 0.50)	(0.90, 0.30)	(0.90, 0.30)	(0.60, 0.60)
	$\Upsilon_{4}$	(0.70, 0.50)	(0.60, 0.60)	(0.90, 0.30)	(0.70, 0.50)	(0.95, 0.25)
	$\Upsilon_{5}$	(0.90, 0.30)	(0.85, 0.35)	(0.80, 0.40)	(0.85, 0.35)	(0.90, 0.30)
	$\Upsilon_{6}$	(0.90, 0.30)	(0.90, 0.30)	(0.95, 0.25)	(0.90, 0.30)	(0.90, 0.30)
*E* ^(3)^	$\Upsilon_{1}$	(0.80, 0.40)	(0.90, 0.30)	(0.80, 0.40)	(0.85, 0.35)	(0.70, 0.50)
	$\Upsilon_{2}$	(0.90, 0.30)	(0.70, 0.50)	(0.85, 0.35)	(0.70, 0.50)	(0.60, 0.60)
	$\Upsilon_{3}$	(0.80, 0.40)	(0.70, 0.50)	(0.60, 0.60)	(0.90, 0.30)	(0.80, 0.40)
	$\Upsilon_{4}$	(0.95, 0.25)	(0.85, 0.35)	(0.95, 0.25)	(0.80, 0.40)	(0.90, 0.30)
	$\Upsilon_{5}$	(0.90, 0.30)	(0.60, 0.60)	(0.90, 0.30)	(0.90, 0.30)	(0.85, 0.35)
	$\Upsilon_{6}$	(0.90, 0.30)	(0.90, 0.30)	(0.90, 0.30)	(0.85, 0.35)	(0.95, 0.25)
*E* ^(4)^	$\Upsilon_{1}$	(0.60, 0.60)	(0.70, 0.50)	(0.90, 0.30)	(0.85, 0.35)	(0.95, 0.25)
	$\Upsilon_{2}$	(0.90, 0.30)	(0.85, 0.35)	(0.70, 0.50)	(0.80, 0.40)	(0.80, 0.40)
	$\Upsilon_{3}$	(0.85, 0.35)	(0.90, 0.30)	(0.90, 0.30)	(0.90, 0.30)	(0.90, 0.30)
	$\Upsilon_{4}$	(0.90, 0.30)	(0.90, 0.30)	(0.90, 0.30)	(0.95, 0.25)	(0.60, 0.60)
	$\Upsilon_{5}$	(0.60, 0.60)	(0.70, 0.50)	(0.85, 0.35)	(0.80, 0.40)	(0.85, 0.35)
	$\Upsilon_{6}$	(0.95, 0.25)	(0.95, 0.25)	(0.95, 0.25)	(0.85, 0.35)	(0.90, 0.30)

**Table 6:** The comprehensive decision matrix obtained by the FFSSWA operator.

Alternative	$C_{1}$	$C_{2}$	$C_{3}$	$C_{4}$	$C_{5}$
$\Upsilon_{1}$	(0.8758, 0.3236)	(0.8455, 0.3531)	(0.8737, 0.3236)	(0.8743, 0.3263)	(0.9008, 0.3115)
$\Upsilon_{2}$	(0.9244, 0.2802)	(0.8674, 0.3324)	(0.7791, 0.4533)	(0.7887, 0.4098)	(0.7635, 0.4304)
$\Upsilon_{3}$	(0.8148, 0.3846)	(0.8154, 0.3771)	(0.9262, 0.2750)	(0.8886, 0.3119)	(0.8325, 0.3637)
$\Upsilon_{4}$	(0.9345, 0.2718)	(0.8404, 0.3565)	(0.9086, 0.3357)	(0.8966, 0.3177)	(0.9192, 0.2875)
$\Upsilon_{5}$	(0.8623, 0.3364)	(0.8167, 0.3778)	(0.8791, 0.3215)	(0.8797, 0.3209)	(0.8864, 0.3142)
$\Upsilon_{6}$	(0.9219, 0.2826)	(0.9388, 0.2648)	(0.9325, 0.2769)	(0.8864, 0.3142)	(0.9376, 0.2662)

**Table 7:** The integration outcomes by utilizing FFSSWA and FFSSWG operators.

Suppliers	Sum Measure ${\tilde{P}}_{s}$ by the FFSSWA Operator	Score $S\left({\tilde{P}}_{s}\right)$	Product Measure ${\tilde{Q}}_{s}$ by the FFSSWG Operator	Score $S\left({\tilde{Q}}_{s}\right)$
$\Upsilon_{1}$	(0.8750, 0.3276)	0.6881	(0.8703, 0.3303)	0.6785
$\Upsilon_{2}$	(0.8444, 0.3598)	0.6272	(0.8032, 0.4031)	0.5524
$\Upsilon_{3}$	(0.8881, 0.3148)	0.7159	(0.8572, 0.3390)	0.6523
$\Upsilon_{4}$	(0.9032, 0.3141)	0.7485	(0.8884, 0.3252)	0.7162
$\Upsilon_{5}$	(0.8701, 0.3298)	0.6782	(0.8608, 0.3371)	0.6595
$\Upsilon_{6}$	(0.9275, 0.2793)	0.8051	(0.9185, 0.2853)	0.7838

**Table 8:** The comprehensive decision matrix obtained by the FFFWA operator.

Suppliers	P$G_{s}^{\left(1\right)}$	Ranking	$G_{s}^{\left(2\right)}$	Ranking	$G_{s}^{\left(3\right)}$	Ranking	${ℜ}_{s}$	Ranking
$\Upsilon_{1}$	0.1645	4	2.3255	4	0.8601	4	1.9570	4
$\Upsilon_{2}$	0.1420	6	2.0000	6	0.7424	6	1.5731	6
$\Upsilon_{3}$	0.1647	3	2.3224	3	0.8611	3	2.1112	3
$\Upsilon_{4}$	0.1764	2	2.4901	2	0.9218	2	2.4130	2
$\Upsilon_{5}$	0.1611	5	2.2754	5	0.8419	5	1.8206	5
$\Upsilon_{6}$	0.1913	1	2.7027	1	1.0000	1	2.7613	1

**Table 9:** The impact of σ on ultimate decision results.

σ	Ranking Values						Sorting
	$ℜ(\Upsilon_{1})$	$ℜ(\Upsilon_{2})$	$ℜ(\Upsilon_{3})$	$ℜ(\Upsilon_{4})$	$ℜ(\Upsilon_{5})$	$ℜ(\Upsilon_{6})$	
σ=−1	2.0811	1.5443	1.9168	2.3444	1.8142	2.7613	$\Upsilon_{6}≻\Upsilon_{4}≻\Upsilon_{1}≻\Upsilon_{3}≻\Upsilon_{5}≻\Upsilon_{2}$
σ=−2	1.9570	1.5731	2.1112	2.4130	1.8206	2.7613	$\Upsilon_{6}≻\Upsilon_{4}≻\Upsilon_{3}≻\Upsilon_{1}≻\Upsilon_{5}≻\Upsilon_{2}$
σ=−5	2.0332	1.6739	2.1915	2.4949	1.8373	2.7613	$\Upsilon_{6}≻\Upsilon_{4}≻\Upsilon_{3}≻\Upsilon_{1}≻\Upsilon_{5}≻\Upsilon_{2}$
σ=−10	2.2536	1.7576	2.0896	2.5281	1.8534	2.7613	$\Upsilon_{6}≻\Upsilon_{4}≻\Upsilon_{1}≻\Upsilon_{3}≻\Upsilon_{5}≻\Upsilon_{2}$
σ=−20	2.3059	1.8019	2.1039	2.5427	1.8614	2.7613	$\Upsilon_{6}≻\Upsilon_{4}≻\Upsilon_{1}≻\Upsilon_{3}≻\Upsilon_{5}≻\Upsilon_{2}$
σ=−50	2.3448	1.8646	2.1042	2.5477	1.8282	2.7613	$\Upsilon_{6}≻\Upsilon_{4}≻\Upsilon_{1}≻\Upsilon_{3}≻\Upsilon_{5}≻\Upsilon_{2}$
σ=−100	2.3582	1.8739	2.1027	2.5487	1.8277	2.7613	$\Upsilon_{6}≻\Upsilon_{4}≻\Upsilon_{1}≻\Upsilon_{3}≻\Upsilon_{5}≻\Upsilon_{2}$

**Table 10:** The impact of σ on ultimate decision results.

σ	Ranking Values						Sorting
	$ℜ(\Upsilon_{1})$	$ℜ(\Upsilon_{2})$	$ℜ(\Upsilon_{3})$	$ℜ(\Upsilon_{4})$	$ℜ(\Upsilon_{5})$	$ℜ(\Upsilon_{6})$	
0.1	1.9599	1.5519	2.0971	2.4093	1.8203	2.7613	$\Upsilon_{6}≻\Upsilon_{4}≻\Upsilon_{3}≻\Upsilon_{1}≻\Upsilon_{5}≻\Upsilon_{2}$
0.2	1.9592	1.5572	2.1007	2.4102	1.8204	2.7613	$\Upsilon_{6}≻\Upsilon_{4}≻\Upsilon_{3}≻\Upsilon_{1}≻\Upsilon_{5}≻\Upsilon_{2}$
0.3	1.9584	1.5625	2.1042	2.4112	1.8205	2.7613	$\Upsilon_{6}≻\Upsilon_{4}≻\Upsilon_{3}≻\Upsilon_{1}≻\Upsilon_{5}≻\Upsilon_{2}$
0.4	1.9577	1.5678	2.1077	2.4121	1.8205	2.7613	$\Upsilon_{6}≻\Upsilon_{4}≻\Upsilon_{3}≻\Upsilon_{1}≻\Upsilon_{5}≻\Upsilon_{2}$
0.5	1.9570	1.5731	2.1112	2.4130	1.8206	2.7613	$\Upsilon_{6}≻\Upsilon_{4}≻\Upsilon_{3}≻\Upsilon_{1}≻\Upsilon_{5}≻\Upsilon_{2}$
0.6	1.9570	1.5731	2.1112	2.4130	1.8206	2.7613	$\Upsilon_{6}≻\Upsilon_{4}≻\Upsilon_{3}≻\Upsilon_{1}≻\Upsilon_{5}≻\Upsilon_{2}$
0.7	1.9563	1.5784	2.1147	2.4139	1.8207	2.7613	$\Upsilon_{6}≻\Upsilon_{4}≻\Upsilon_{3}≻\Upsilon_{1}≻\Upsilon_{5}≻\Upsilon_{2}$
0.8	1.9548	1.5888	2.1216	2.4158	1.8208	2.7613	$\Upsilon_{6}≻\Upsilon_{4}≻\Upsilon_{3}≻\Upsilon_{1}≻\Upsilon_{5}≻\Upsilon_{2}$
0.9	1.9541	1.5939	2.1250	2.4167	1.8209	2.7613	$\Upsilon_{6}≻\Upsilon_{4}≻\Upsilon_{3}≻\Upsilon_{1}≻\Upsilon_{5}≻\Upsilon_{2}$
1.0	1.9534	1.5990	2.1284	2.4176	1.8210	2.7613	$\Upsilon_{6}≻\Upsilon_{4}≻\Upsilon_{3}≻\Upsilon_{1}≻\Upsilon_{5}≻\Upsilon_{2}$

**Table 11:** The impact of different weight types for the ultimate decision results.

Weight Type	Ranking Values						Sorting
	$ℜ(\Upsilon_{1})$	$ℜ(\Upsilon_{2})$	$ℜ(\Upsilon_{3})$	$ℜ(\Upsilon_{4})$	$ℜ(\Upsilon_{5})$	$ℜ(\Upsilon_{6})$	
Objective weight	2.1022	1.6187	1.7914	2.4117	1.8975	2.7613	$\Upsilon_{6}≻\Upsilon_{4}≻\Upsilon_{1}≻\Upsilon_{5}≻\Upsilon_{3}≻\Upsilon_{2}$
Subjective weight	1.9698	1.5368	2.1676	2.4237	1.8494	2.7613	$\Upsilon_{6}≻\Upsilon_{4}≻\Upsilon_{3}≻\Upsilon_{1}≻\Upsilon_{5}≻\Upsilon_{2}$
Combinative weight	1.9570	1.5731	2.1112	2.4130	1.8206	2.7613	$\Upsilon_{6}≻\Upsilon_{4}≻\Upsilon_{3}≻\Upsilon_{1}≻\Upsilon_{5}≻\Upsilon_{2}$
Equal weight	2.1118	1.6383	1.8034	2.4379	1.9124	2.7613	$\Upsilon_{6}≻\Upsilon_{4}≻\Upsilon_{1}≻\Upsilon_{3}≻\Upsilon_{5}≻\Upsilon_{2}$

**Table 12:** The impact of different weight types for the ultimate decision results.

Approaches	Ranking Values						Sorting
	$ℜ(\Upsilon_{1})$	$ℜ(\Upsilon_{2})$	$ℜ(\Upsilon_{3})$	$ℜ(\Upsilon_{4})$	$ℜ(\Upsilon_{5})$	$ℜ(\Upsilon_{6})$	
FF-TOPSIS method proposed by [27]	0.5776	0.1602	0.5383	0.7430	0.5241	0.9676	$\Upsilon_{6}≻\Upsilon_{4}≻\Upsilon_{1}≻\Upsilon_{3}≻\Upsilon_{5}≻\Upsilon_{2}$
FF-WASPAS method proposed by [30]	0.6274	0.4842	0.6190	0.6821	0.6100	0.7605	$\Upsilon_{6}≻\Upsilon_{4}≻\Upsilon_{1}≻\Upsilon_{3}≻\Upsilon_{5}≻\Upsilon_{2}$
FF-WPM method proposed by [28]	0.6255	0.4687	0.6059	0.6754	0.6059	0.7560	$\Upsilon_{6}≻\Upsilon_{4}≻\Upsilon_{1}≻\Upsilon_{3}≻\Upsilon_{5}≻\Upsilon_{2}$
FF-VIKOR method proposed by [31]	0.5123	1.0000	0.6255	0.4219	0.6296	0.0000	$\Upsilon_{6}≻\Upsilon_{4}≻\Upsilon_{1}≻\Upsilon_{3}≻\Upsilon_{5}≻\Upsilon_{2}$
FF-ARAS method proposed by [31]	0.8143	0.6464	0.8180	0.8913	0.7947	0.9898	$\Upsilon_{6}≻\Upsilon_{4}≻\Upsilon_{3}≻\Upsilon_{1}≻\Upsilon_{5}≻\Upsilon_{2}$
FF-SAW method proposed by [31]	0.6293	0.4996	0.6322	0.6889	0.6142	0.7650	$\Upsilon_{6}≻\Upsilon_{4}≻\Upsilon_{3}≻\Upsilon_{1}≻\Upsilon_{5}≻\Upsilon_{2}$
FF-CoCoSo method in this study	1.9570	1.5731	2.1112	2.4130	1.8206	2.7613	$\Upsilon_{6}≻\Upsilon_{4}≻\Upsilon_{3}≻\Upsilon_{1}≻\Upsilon_{5}≻\Upsilon_{2}$

**Table 13:** Characteristic comparison between the propounded method and other Fermatean fuzzy decision algorithms.

Methods	Calculation of Expert Weights	Flexibility of the Fusion Procedure	Criteria Weights	Ranking Algorithm	Considers Multiple Fusion Strategies
FF-TOPSIS method proposed by [27]	Assume	NO	Subjective	TOPSIS	NO
FF-WASPAS method proposed by [30]	Computing	NO	Objective	WASPAS	NO
FF-WPM method proposed by [28]	NO	NO	Subjective	WPM	NO
FF-VIKOR method proposed by [31]	NO	NO	Subjective	VIKOR	NO
FF-ARAS method proposed by [31]	NO	NO	Subjective	ARAS	NO
FF-SAW method proposed by [31]	NO	NO	Subjective	SAW	NO
The propounded method in this study	Computing	YES	Combined weight	CoCoSo	YES

## Data Availability

The data presented in this study are available in this article.

## Abbreviations

The abbreviations displayed in the following are used in this manuscript.

Fermatean Fuzzy Set	FFS
Combined Compromise Solution	CoCoSo
Multi-Criteria Group Decision-Making	MCGDM
Best-and-Worst Method	BWM
Fuzzy Set	FS
Non-Membership Grade	NMG
Membership Grade	MG
Fermatean Fuzzy Number	FFN
Weighted Product Model	WPM
Simple Additive Weighting	SAW
Additive Ratio ASsessment	ARAS
VIse KriterijumsaOptimiz acija I Kompromisno Resenje	VIKOR
CRiteria Importance Through Intercriteria Correlation	CRITIC
Evaluation based on Distance from Average Solution	EDAS
An acronym in Portuguese of interactive and multi-criteria decision making	TODIM
Multi-objective optimization based on the ratio analysis with the full multiplicative form	MULTIMOORA
COmplex PRoportional ASsessment	COPRAS
Technique for Order Preference by Similarity to an Ideal Solution	TOPSIS
Fermatean Fuzzy Schweizer–Sklar Weighted Averaging	FFSSWA
Fermatean Fuzzy Schweizer–Sklar Weighted Geometric	FFSSWG

The notions and their explanations are displayed in the following.

$\phi_{F}\left(y\right)$	Membership function
$\psi_{F}\left(y\right)$	Non-membership function
F	Fermatean fuzzy number
S	Score function of FFN F
$E\left(F\right)$	Entropy of FFN F
${\tilde{T}}_{SS,\sigma }\left(a,b\right)$	Schweizer–Sklar T-norm
${\tilde{T}}_{SS,\sigma }^{*}\left(a,b\right)$	Schweizer–Sklar S-norm
${\bar{F}}^{l}={\left({\bar{F_{st}}}^{l}\right)}_{m\times n}$	Decision matrix of expert
$\Upsilon_{s}$	The *s*th alternative
$C_{t}$	The *t*th criteria
$\omega_{t}$	Weight of *t*th criteria
$\nu_{1}$	Weight of *l*th expert
$F^{l}={\left(F_{st}^{l}\right)}_{m\times n}$	Normalized Fermatean fuzzy assessment matrices
$\omega_{t}^{obj}$	The objective Weight of *t*th criteria
$\omega_{t}^{sub}$	The subjective Weight of *t*th criteria
${\tilde{P}}_{s}$	FFSSWA comparability sequence
${\tilde{Q}}_{s}$	FFSSWG comparability sequence
$G_{s}^{\left(1\right)}$	Arithmetic mean strategy score
$G_{s}^{\left(2\right)}$	Relative score strategy
$G_{s}^{\left(3\right)}$	Balanced compromise strategy score
ϱ	Balancing coefficient in $G_{s}^{\left(3\right)}$
${\tilde{R}}^{\left(y\right)}$	Rank of supplier $\Upsilon_{s}$ by $G_{s}^{\left(y\right)}$
